# Replicative age differentially predicts metabolic competence across industrial yeasts at single-cell resolution

**DOI:** 10.1093/femsyr/foag037

**Published:** 2026-08-07

**Authors:** Marco Eigenfeld, Elisabeth Zach, Ann-Kathrin Bürkle, Benjamin Schneider, Sebastian P Schwaminger

**Affiliations:** Medical University of Graz, Otto Loewi Research Center, Division of Medicinal Chemistry, NanoLab; Neue Stiftingtalstraße 6, 8010, Graz, Austria; BioTechMed-Graz, Mozartgasse 12/II, 8010, Graz, Austria; Medical University of Graz, Otto Loewi Research Center, Division of Medicinal Chemistry, NanoLab; Neue Stiftingtalstraße 6, 8010, Graz, Austria; Medical University of Graz, Otto Loewi Research Center, Division of Medicinal Chemistry, NanoLab; Neue Stiftingtalstraße 6, 8010, Graz, Austria; Medical University of Graz, Otto Loewi Research Center, Division of Medicinal Chemistry, NanoLab; Neue Stiftingtalstraße 6, 8010, Graz, Austria; Medical University of Graz, Otto Loewi Research Center, Division of Medicinal Chemistry, NanoLab; Neue Stiftingtalstraße 6, 8010, Graz, Austria; BioTechMed-Graz, Mozartgasse 12/II, 8010, Graz, Austria

**Keywords:** flow cytometry, replicative aging, bud scars, mCherry, CFDA, industrial fermentation

## Abstract

In industrial yeast fermentations, population-level viability assays routinely report healthy cultures even as aged, metabolically compromised mother-cell subpopulations go undetected. Replicative age contributes to this hidden heterogeneity, but its link to a cell’s metabolic competence has been difficult to measure simultaneously in the same cell by high-throughput flow cytometry. Here, we introduce a dual-parameter flow cytometry workflow combining bud-scar labeling with a recombinant His6–SUMO–mCherry–chitin-binding domain fusion protein (∼610 nm emission) and 5(6)-carboxyfluorescein diacetate (CFDA)-based viability detection (∼525 nm), providing spectral orthogonality without computational autofluorescence correction. We applied it to four industrially relevant yeasts: *Saccharomyces pastorianus* W-34/70, *S. cerevisiae* var. *diastaticus* BE-134, *S. cerevisiae* var. *chevalieri* LA-01, and *Komagataella phaffii* X33. In *S. pastorianus*, viability declined monotonically from 88% in daughter cells to 5% in the oldest resolved mother-cell class; longitudinal monitoring over 96 h captured a progressive widening of the age-dependent viability gradient masked at the population level. Among *S. cerevisiae* variants, age-dependent gradients were weaker and strain-specific, while in *K. phaffii* reliable discrimination required exponential growth. Replicative age is thus a strain-dependent predictor of metabolic competence rather than a universally conserved one, and the workflow offers brewers and bioprocess developers a practical tool for age-resolved fermentation monitoring and pitching-yeast assessment.

## Teaser

A dual-staining technique now measures both age and vitality of individual yeast cells, helping brewers and bioproducers spot aging subpopulations that weaken fermentations but remain invisible to standard tests.

## Introduction

The performance and stability of yeast populations in industrial fermentations depend critically on how physiological heterogeneity emerges and evolves during growth. A major biological driver of this heterogeneity is replicative aging: during asymmetric cell division, mother cells accumulate bud scars and undergo progressive changes in metabolic activity, stress tolerance, and growth capacity, whereas newly formed daughter cells are rejuvenated (Henderson et al. 2014). Even within genetically identical populations, replicative age therefore generates pronounced single-cell variability in growth rate, metabolic output, and survival (Knorre et al. 2018, Eigenfeld and Schwaminger 2025).

These age-associated changes carry direct consequences for industrial bioprocesses. Altered substrate uptake, reduced biomass formation, and increased sensitivity to environmental stressors have all been linked to age-dependent phenotypes in fermenting yeast populations, ultimately contributing to process variability and performance loss (Eigenfeld et al. 2021b, 2023b). The practical relevance of these effects differs strongly between application contexts. In brewing, repitching reuses yeast cropped from a specific flocculated or sedimented fraction of one fermentation as inoculum for the next, and therefore does not sample the population at random. Cell size, flocculation behavior, and sedimentation rate are correlated with replicative age. This nonrandom harvesting can therefore selectively retain older mother cells and enrich successive batches for aged subpopulations, a pattern associated with shifts in fermentation rate, attenuation, and flavor-relevant metabolite profiles (Powell et al. 2003b, Eigenfeld et al. 2021b, 2023b, Kuřec et al. 2009). This enrichment is a consequence of selective cropping rather than of serial passaging per se: random sampling of a freely growing population would instead preserve the geometric age distribution that arises from asymmetric division (Hartwell and Unger 1977). Consistent with this, serial repitching does not inevitably lead to progressive aging: whether older mother cells accumulate depends on process-specific factors such as size-dependent settling in the fermenter cone and the harvesting regime, and stable rejuvenation across many runs has been documented in industrial lager fermentations (Bühligen et al. 2014). In recombinant protein production with nonconventional hosts such as *Komagataella phaffii*, age-heterogeneous populations under prolonged fed-batch conditions pose challenges for yield consistency and product quality (Gangl et al. 2025). Despite this practical relevance, lineage history is not currently assessed as part of routine pitching-yeast quality control or bioprocess monitoring, because available analytical methods do not deliver age-resolved viability data with the throughput and ease of use required in applied settings (Eigenfeld and Schwaminger 2025). Understanding how replicative age shapes population behavior is therefore not only a fundamental biological question but also an unsolved practical challenge for strain selection and process optimization (Eigenfeld and Schwaminger 2025).

Since the seminal observation by Mortimer and Johnston that individual yeast cells possess a finite replicative lifespan (Mortimer and Johnston 1959), the mechanistic basis of replicative aging in *S. cerevisiae* has been extensively characterized, with established contributions from mitochondrial dysfunction, vacuolar acidification, loss of protein homeostasis, and asymmetric segregation of damaged cellular components between mother and daughter cells (Henderson et al. 2014, Sorokin et al. 2014, Knorre et al. 2018, Yang et al. 2022). At the systems level, integrated proteome and transcriptome studies across the replicative lifespan have identified the uncoupling of protein biogenesis machinery from its transcript regulation as a central driver of aging, accompanied by metabolic shifts and the loss of stoichiometry in protein complexes (Janssens et al. 2015, Leupold et al. 2019). More recent single-cell biosensor approaches have further established physicochemical hallmarks such as cytosolic acidification and altered macromolecular crowding as quantifiable signatures of yeast aging (Henderson et al. 2014, Mouton et al. 2020). Replicative aging is also conserved beyond the *Saccharomyces* lineage and has been documented in pathogenic yeasts such as *Candida glabrata, Candida auris*, and *Cryptococcus neoformans*, where it modulates antifungal resistance and virulence (Bhattacharya et al. 2020). Methodologically, microfluidic single-cell trapping platforms have transformed lifespan analysis by enabling continuous tracking of individual mother cells throughout their entire replicative lifespan with high throughput (Zhang et al. 2012, Crane et al. 2014, Jo et al. 2015), complementing classical approaches such as micromanipulation, density-based separation, biotinylation, and magnetic isolation of bud-scar-labeled mother cells (Eigenfeld et al. 2023b, Wittmann et al. 2024).

Despite this progress, the extent to which the relationship between replicative age and physiological competence is conserved across industrially relevant yeast strains and species remains poorly understood. Most mechanistic insights derive from a small number of laboratory *S. cerevisiae* strains, whereas industrial fermentations increasingly rely on a diverse panel of yeasts with distinct metabolic strategies, and natural variation in aging phenotypes across *S. cerevisiae* isolates has been documented even within the species, specifically as strain-dependent variation in chronological lifespan, (Jung et al. 2018). Classical lager strains such as *Saccharomyces pastorianus* are characterized by pronounced mother–daughter asymmetry and well-documented replicative aging phenotypes (Eigenfeld et al. 2021b, 2023b, Leupold et al. 2019), whereas metabolically specialized *S. cerevisiae* variants represent fundamentally different metabolic programs. *S. cerevisiae* var. *diastaticus* is a hyperattenuating strain capable of fermenting residual dextrins via extracellular glucoamylase activity encoded by the *STA1* gene, and has been reevaluated as both a spoilage organism and a legitimate production strain in specialty brewing (Meier-Dörnberg et al. 2018, Krogerus and Gibson 2020). *Saccharomyces cerevisiae* var. *chevalieri* is a maltose-negative strain incapable of fermenting the dominant wort sugar, and is specifically employed for the biological production of low- and nonalcoholic beers (Simon et al. 2024, Pater et al. 2024). Nonconventional hosts such as *K. phaffii* further extend this diversity with distinct methylotrophic carbon metabolism (Bernauer et al. 2020) and are increasingly engineered as cell factories for heterologous production (Cheng et al. 2025), and although replicative aging has been comparatively little studied in this species, recent work points to species-specific viability and stress-response signatures that diverge from the *Saccharomyces* paradigm (Gangl et al. 2025). Whether age-dependent changes in metabolic competence are a conserved feature of budding yeasts or a strain- and species-specific phenomenon has not been systematically addressed.

Resolving this question requires analytical approaches that can simultaneously quantify replicative age and physiological state at single-cell resolution. Yeast viability and vitality have traditionally been assessed by colony-forming-unit counting and methylene-blue staining, both of which suffer from well-documented limitations: CFU enumeration requires 24–48 h and captures only proliferative capacity, while methylene blue underestimates damaged-but-viable subpopulations and depends strongly on dye purity (Kwolek-Mirek and Zadrag-Tecza 2014). Flow cytometry has therefore emerged as the dominant high-throughput platform for yeast physiological profiling, supporting a broad palette of fluorogenic probes that report on complementary aspects of cellular state (Eigenfeld et al. 2023a, Davey and Kell 1996, Deere et al. 1998). Membrane integrity is most commonly assessed by exclusion of propidium iodide or with combined SYTO 9/PI live–dead kits; intracellular esterase activity and metabolic competence are reported by fluorescein diacetate (FDA), 5(6)-carboxyfluorescein diacetate (CFDA), or FUN-1 (Eigenfeld et al. 2023a, Diaper and Edwards 1994, Breeuwer et al. 1995, Weigert et al. 2009); and intracellular pH has been resolved both by short-incubation CFDA-based protocols and by quantitative fluorescent biosensors (Bracey et al. 1998, Rennick et al. 2022). Conceptually, these probes fall into two complementary categories: *viability* markers, which report whether a cell is alive in the sense of possessing an intact membrane, and *vitality* markers, which report on physiological capacity beyond mere survival (Kwolek-Mirek and Zadrag-Tecza 2014). CFDA-derived fluorescence is widely used in brewing and bioprocess contexts because it integrates membrane integrity with active enzymatic conversion and thereby reports on vitality rather than mere viability (Eigenfeld et al. 2023a, Weigert et al. 2009).

Replicative age, in turn, can be inferred from the chitin-rich bud scars that persist on the mother-cell surface after each cytokinesis, using calcofluor-type chitin-binding dyes, wheat-germ-agglutinin (WGA) conjugates, or recombinant fluorescent protein linkers carrying a chitin-binding domain (Powell et al. 2003a, Eigenfeld et al. 2021a). WGA-based bud-scar labeling has been adapted to flow cytometry and applied to monitor cell-age distributions in industrial beer fermentations, demonstrating the principal feasibility of high-throughput cytometric age assessment (Kuřec et al. 2009). In parallel, multiparameter flow cytometry workflows that combine viability staining with markers for membrane potential, mitochondrial activity, glycogen content, and intracellular pH have been established for fermentation monitoring (Sommer 2020), providing a rich physiological readout at single-cell resolution.

Despite this methodological maturity, replicative age and metabolic competence have to date been measured in separate assays rather than in the same cell, and no flow-cytometric workflow has been reported that simultaneously resolves both readouts at single-cell resolution. We emphasise that replicative-age determination itself is long established: the finite replicative lifespan of individual cells was first demonstrated by micromanipulation (Mortimer and Johnston 1959), and bud-scar age has since been quantified by calcofluor- and WGA-based staining, biotinylation and magnetic sorting, mother-enrichment strategies, and microfluidic mother-cell tracking, including WGA-based flow cytometry of industrial fermentations (Powell et al. 2003a, Eigenfeld et al. 2023b, Kuřec et al. 2009, Crane et al. 2014). The advance reported here is therefore not age measurement per se, but the spectral relocation of the bud-scar signal to a red channel, which for the first time renders replicative age and CFDA-based metabolic competence simultaneously measurable in the same cell. This separation is not incidental: most established bud-scar markers rely on green fluorophores (e.g. FITC, calcofluor, GFP, and Alexa Fluor 488–WGA) that spectrally overlap with CFDA- and FDA-based viability and vitality readouts as well as with intrinsic yeast autofluorescence, precluding robust multiparameter analysis without computational unmixing (Maslanka et al. 2018, Eigenfeld et al. 2022). As a direct consequence, the relationship between an individual cell’s lineage history and its physiological state has remained inaccessible to high-throughput cytometric analysis; population-level correlations have instead been inferred either from age-fractionated samples obtained by magnetic separation or elutriation (Eigenfeld et al. 2023b), or from low-throughput microfluidic lifespan tracking (Crane et al. 2014, Jo et al. 2015). A bud-scar probe whose emission lies in a spectrally distinct red channel would close this methodological gap by enabling direct, simultaneous, single-cell readout of replicative age and metabolic competence within the same cell, combining the throughput of conventional flow cytometry with the lineage resolution previously restricted to microfluidic platforms.

Here, we address both the methodological and biological gaps outlined above. We introduce a red-channel bud-scar labeling strategy based on a chitin-binding domain fused to mCherry (His_6_–SUMO–mCherry–ChBD), placing the age-associated fluorescence in a spectrally distinct ECD channel and enabling simultaneous flow-cytometric quantification of replicative age and metabolic viability in individual cells without spectral interference or offline correction. Applying this framework to a panel of four industrially relevant yeast strains—*S. pastorianus* W-34/70 (SafLager W-34/70, Fermentis), *S. cerevisiae* var. *diastaticus* BE-134 (SafAle BE-134, Fermentis), *S. cerevisiae* var. *chevalieri* LA-01 (SafBrew LA-01, Fermentis), and *K. phaffii* X33—we investigate how the coupling between replicative age and metabolic competence varies across strains and species with distinct metabolic strategies. In doing so, we establish a framework for lineage-resolved single-cell analysis of yeast population physiology and demonstrate that replicative age is a strain-dependent, rather than universally conserved, predictor of metabolic competence.

## Materials and methods

### Construction of the mCherry–ChBD fusion protein

The gene encoding mCherry was amplified from the pET MBP–mCherry LIC vector (Addgene plasmid #29747) and inserted into a modified pET28b(+) expression vector containing an N-terminal His_6_–SUMO tag and a chitin-binding domain (ChBD) derived from *Bacillus circulans* using Gibson assembly, resulting in the construct His_6_–SUMO–mCherry–ChBD. The ligation product was transformed into chemically competent *Escherichia coli* NEB10-beta (New England Biolabs) for cloning and plasmid propagation. Correct insertion and reading frame integrity were verified by colony polymerase chain reaction (PCR) using T7 promoter primers followed by Sanger sequencing of positive clones. One sequence-verified clone was selected for plasmid preparation and subsequent transformation into the expression host. Detailed plasmid maps and primer sequences are provided in Table S1.

### Heterologous protein expression

The fusion protein was heterologously expressed in *E. coli* BL21(DE3). Precultures were grown overnight in Lennox LB medium supplemented with kanamycin (50 μg ml⁻¹) and used to inoculate main cultures to an initial optical density at 600 nm (OD_600_) of 0.1. Cultures were incubated at 37°C with shaking at 160 rpm until an OD_600_ of 0.6–0.8 was reached. Protein expression was induced by addition of isopropyl β-d-1-thiogalacto pyranoside to a final concentration of 0.5 mM, followed by incubation at 25°C overnight. Cells were harvested by centrifugation (4000 × *g*, 10 min, 4°C) and stored at −20°C until further processing.

### Protein purification and concentration determination

Purification of the His_6_–SUMO–mCherry–ChBD fusion protein was performed according to a previously established protocol (Eigenfeld et al. 2021b). Briefly, cell pellets were resuspended in lysis buffer and disrupted by sonication. Sonication was performed in two cycles of 5 min each (1 s on/1 s off, 40% amplitude), with a 1 min pause on ice between cycles to prevent sample overheating. The lysate was clarified by centrifugation (4000 × *g*, 30 min, 4°C) and the resulting supernatant was subjected to immobilized metal affinity chromatography (HisTrap excel, Cytiva) to achieve sufficient purity (Table S2). Purified protein fractions were dialyzed overnight against 20 mM MOPS buffer (pH 7.4). Protein purity and molecular weight were verified by Tricine sodium dodecyl sulfate–polyacrylamide gel electrophoresis (SDS–PAGE). Protein concentration was determined spectrophotometrically using the Beer–Lambert law. The extinction coefficient was obtained by comparing absorbance at 280 nm of samples diluted in native buffer and in 6 M urea, assuming equal protein concentrations in both conditions.

### Yeast strains and cultivation

Four industrially relevant yeast strains were selected to represent a range of metabolic strategies in budding yeasts. *Saccharomyces pastorianus* W-34/70 (SafLager W-34/70, Fermentis) served as the primary model strain for method development and validation, representing a classical lager yeast with well-characterized replicative aging and pronounced mother–daughter asymmetry (Eigenfeld et al. 2021a, 2023b). *Saccharomyces cerevisiae* var. *diastaticus* BE-134 (SafAle BE-134, Fermentis) is a hyperattenuating strain carrying the *STA1* gene, which encodes an extracellular glucoamylase enabling fermentation of residual dextrins and starch (Meier-Dörnberg et al. 2018, Krogerus and Gibson 2020). *Saccharomyces cerevisiae* var. *chevalieri* LA-01 (SafBrew LA-01, Fermentis) is a maltose- and maltotriose-negative strain specifically developed for the biological production of low- and nonalcoholic beverages (Simon et al. 2024, Pater et al. 2024). *Komagataella phaffii* X33 represents a nonconventional methylotrophic production host with distinct carbon metabolism and stress-response characteristics (Gangl et al. 2025, Cheng et al. 2025).

All strains were maintained on yeast extract–peptone–dextrose (YPD) agar plates and cultivated in liquid YPD medium at 30°C with orbital shaking at 160 rpm. For flow cytometric analysis, overnight cultures were grown for 12–14 h. The following morning, OD_600_ was measured and cultures were diluted with fresh medium to OD_600_ = 1.0 to ensure a comparable number of cells per volume across all strains and to provide equivalent binding sites for the mCherry–ChBD fusion protein. Cells were then harvested by centrifugation (3000 × *g*, 5 min), washed twice with the respective staining buffer, and immediately subjected to staining. All experiments were performed in three independent biological replicates. For *K. phaffii* X33, CFDA-based viability staining was performed on overnight cultures with an OD_600_ of 4.5 ± 0.3, corresponding to late exponential growth phase as confirmed by continued biomass accumulation throughout the cultivation period. Measurements performed on cultures that had reached stationary phase yielded unreliable discrimination between viable and nonviable populations and were excluded from analysis.

### Bud-scar staining with mCherry–ChBD

Bud scars were labeled using the recombinantly produced His_6_–SUMO–mCherry–ChBD fusion protein as described previously with minor modifications (Eigenfeld et al. 2021a, 2022). Yeast cells were adjusted to OD_600_ = 1.0 in staining buffer, and 50 μl of the cell suspension was mixed with 200 μl of a 5000-nM protein solution. Samples were incubated for 30 min at room temperature in the dark with gentle agitation to allow binding of the chitin-binding domain to bud-scar chitin structures. Subsequently, cells were washed twice by centrifugation (3000 × *g*, 5 min) with the corresponding buffer to remove unbound protein. Stained cells were either directly subjected to flow cytometric analysis or processed further for CFDA-based viability staining. Buffer composition and pH conditions were systematically varied as described below to identify optimal conditions for simultaneous age and viability measurements.

### CFDA-based viability staining

Cell viability was assessed using 5(6)-CFDA as an esterase-dependent fluorescent probe (Eigenfeld et al. 2023a). Following bud-scar staining, cells were washed twice by centrifugation (3000 × *g*, 5 min) and resuspended in intracellular pH (ICP) buffer or MOPS buffer adjusted to defined pH values (pH 3–7). CFDA was added at a volume ratio of 1 μl per milliliter of buffer suspension from a 10 mM stock solution, and samples were incubated for 10 min at room temperature in the dark. Intracellular esterases convert nonfluorescent CFDA into fluorescent carboxyfluorescein, which accumulates in metabolically active cells with intact membranes. After incubation, samples were immediately subjected to flow cytometric analysis or confocal laser scanning microscopy (CLSM) without further washing to preserve the intracellular fluorescence signal. Control samples without CFDA were included in each experiment to assess background fluorescence and buffer-dependent autofluorescence. All staining procedures were performed under identical conditions across biological replicates to ensure comparability between buffer systems and pH values. Detailed buffer compositions are provided in Table S3.

### CLSM

To validate simultaneous detection of bud-scar labeling and CFDA-derived metabolic fluorescence at the single-cell level, double-stained yeast samples were analyzed by CLSM. Cells were stained sequentially with His_6_–SUMO–mCherry–ChBD and CFDA as described above and imaged immediately after staining to preserve intracellular CFDA-derived fluorescence. Imaging was performed under ICP buffer conditions at pH 4, which had been identified as a robust condition for combined age and viability readout.

Confocal microscopy was performed using a Nikon Ti Eclipse A1 confocal microscope (Nikon Europe B.V., Amstelveen, the Netherlands) located at the Core Facility Bioimaging, Medical University of Graz. For dual-fluorescence imaging, CFDA-derived fluorescence was excited using the 488 nm laser line, whereas mCherry fluorescence was excited using the 561 nm laser line. The microscope system was additionally equipped with 409 nm, 457/477/514 nm, and 641 nm laser lines, which were not used for image acquisition.

Fluorescence and transmitted-light images were acquired using NIS-Elements AR software (version 4.60.00, 64-bit). CFDA-derived fluorescence was recorded in the green channel, mCherry fluorescence in the red channel, and transmitted-light images were acquired to visualize the total cell population. Images were used for qualitative validation of the dual-staining workflow by confirming mCherry–ChBD localization at bud-scar structures and simultaneous detection of CFDA-derived fluorescence in metabolically active cells.

### Flow cytometry acquisition and gating

Flow cytometric measurements were performed using a benchtop flow cytometer equipped with 488 nm and 561 nm excitation lasers. Fluorescence signals were collected in the FITC channel for CFDA-derived fluorescence and in the ECD channel for mCherry-labeled bud scars. The PerCP channel was used as an autofluorescence reference for viability ratio calculation. Cell size was assessed by forward scatter height (FSC-H) and granularity by side scatter area (SSC-A). For each sample, a minimum of 20 000 events within the yeast-specific gate were recorded. All measurements were performed in three independent biological replicates, each measured in technical triplicate.

Event selection was performed in two sequential steps. First, two-dimensional rectangular gating was applied in FSC-H versus PC7-H parameter space using fixed thresholds (FSC-H: 5 × 10^5^ to 1 × 10^7^; PC7-H: 5 × 10^1^ to 5 × 10^4^). For MOPS buffer at pH 4 and pH 5, a markedly elevated PC7-H autofluorescence was observed (median ~50-fold higher than at pH 6–7), which would result in the exclusion of 86% and 52% of events, respectively under the default gate. For these two conditions, the gate was therefore widened (FSC-H: 1 × 10^6^ to 5 × 10^6^; PC7-H: 5 × 10^3^ to 1 × 10^6^) to retain the full event population. This effect was absent in ICP buffer across all tested pH values and in MOPS buffer at pH 6–7, suggesting a pH-dependent interaction between MOPS buffer and the yeast cell wall. Representative gating plots for both the default and the extended workflow are provided in Fig. S2.

Second, multivariate outlier removal was performed on the pregated population using the Mahalanobis distance calculated from the joint FSC-H and PC7-H distributions. Events exceeding the 99.9th percentile of the χ^2^ distribution (degrees of freedom = 2) were excluded to obtain a homogeneous population for downstream fluorescence analysis. Gating boundaries and outlier thresholds were applied identically across all samples within each buffer and pH condition.

### Data processing and statistical analysis

Flow cytometry data were exported in FCS format and processed using custom analysis scripts implemented in Python (version 3.10). All filtering steps described in the section “Flow cytometry acquisition and gating” were applied identically to all biological replicates and experimental conditions.

Viability was quantified on an event-by-event basis using the ratio of CFDA-derived fluorescence (FITC-A channel) to the autofluorescence reference (PerCP-A channel), hereafter referred to as the CFDA/PerCP ratio. Fixed ratio thresholds were applied per strain group, defined by the position of the bimodal valley in the log_10_(CFDA/PerCP) distribution at pH 3, where viable and nonviable populations exhibit maximal separation. A threshold ratio of 0.7 was applied to *S. pastorianus* W-34/70 and *K. phaffii* X33, and a threshold ratio of 10.0 was applied to *S. cerevisiae* var. *diastaticus* BE-134 and *S. cerevisiae* var. *chevalieri* LA-01 (Fig. S1). The ~10-fold difference in threshold values between the two groups likely reflects strain-specific differences in intracellular esterase activity, membrane permeability, and PerCP autofluorescence. Threshold selection was validated by visual inspection of bimodal distributions across all replicates and pH values and applied consistently across all datasets.

Statistical results are reported as mean ± standard deviation (SD) of biological triplicates unless stated otherwise. Data visualization and statistical summaries were generated using the Python packages NumPy, SciPy, and Matplotlib.

### Age-distribution modeling (Gaussian mixture model)

Replicative age distributions were inferred from mCherry–ChBD fluorescence intensity in the ECD channel using Gaussian mixture modeling (GMM). Fluorescence histograms were fitted with a custom GMM implementation incorporating a minimum peak separation constraint (D_min_ = 500 fluorescence units) to ensure biologically meaningful decomposition into discrete age classes corresponding to daughter cells and successive mother-cell generations. The number of Gaussian components K was selected from the range K = 2–6 based on goodness-of-fit (*R*^2^) to the fluorescence histogram, subject to the constraint that the dominant low-fluorescence peak corresponding to daughter cells carried a minimum component weight of 0.45 to prevent overfitting to rare subpopulations. Model parameters (means, variances, and component weights) were extracted for each biological replicate to quantify shifts in population age structure under different buffer and pH conditions.

Only models fulfilling predefined quality criteria, namely monotonic increases in component means and sufficient separation between adjacent peaks, were accepted for further analysis. Datasets failing these criteria were excluded from age-structure interpretation. Component-specific viability was calculated by combining the age-class assignments from the GMM with event-level CFDA-based viability classification, enabling direct correlation of replicative age and physiological state at single-cell resolution. Full GMM parameters for all strains and conditions are reported in Table S4.

## Results

### Red-channel bud-scar labeling enables simultaneous detection of replicative age and metabolic viability

**Figure 1:**
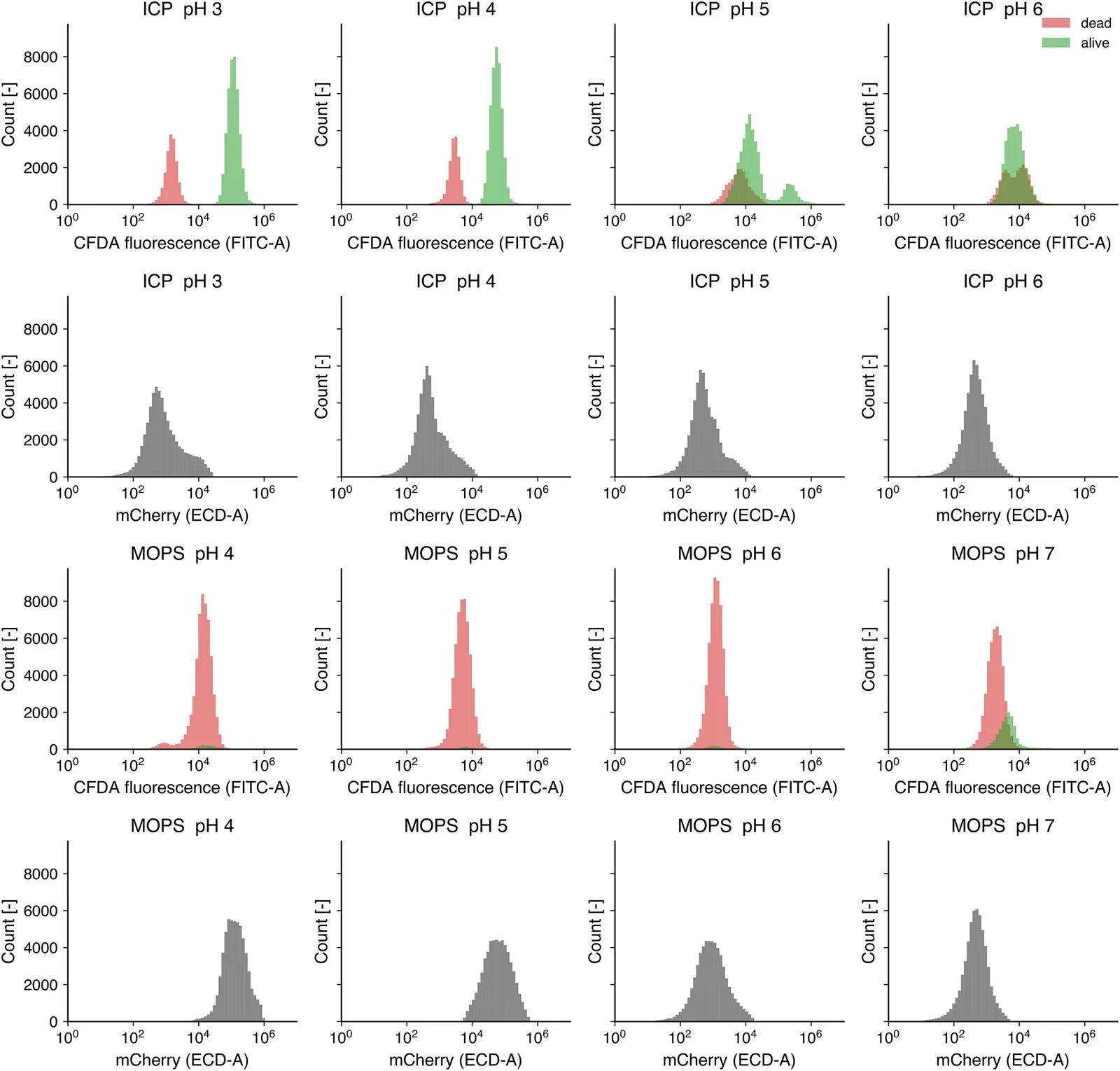
Fluorescence channel distributions of *S. pastorianus* W-34/70 across buffer and pH conditions. **(Rows 1 and 3)** CFDA-derived fluorescence (FITC-A channel) in ICP buffer at pH 3–6 and in MOPS buffer at pH 4–7, with the viable (green) and non-viable (red) populations overlaid. **(Rows 2 and 4)** mCherry–ChBD fluorescence (ECD-A channel) under the same conditions. Representative distributions from one of three biological replicates.

To enable simultaneous flow-cytometric quantification of replicative age and metabolic viability, we combined bud-scar labeling using the His_6_–SUMO–mCherry–ChBD fusion protein, read out in the red ECD channel, with CFDA-based viability staining in the FITC channel. The mCherry signal was detected exclusively in the ECD channel, while CFDA-derived fluorescence was confined to the FITC channel, confirming spectral orthogonality between the two readouts (Fig. 1A and B). Control samples stained with mCherry–ChBD alone showed no detectable signal in the FITC channel, and CFDA staining did not alter ECD fluorescence, demonstrating the absence of spectral crosstalk between age- and viability-associated readouts.

**Figure 2:**
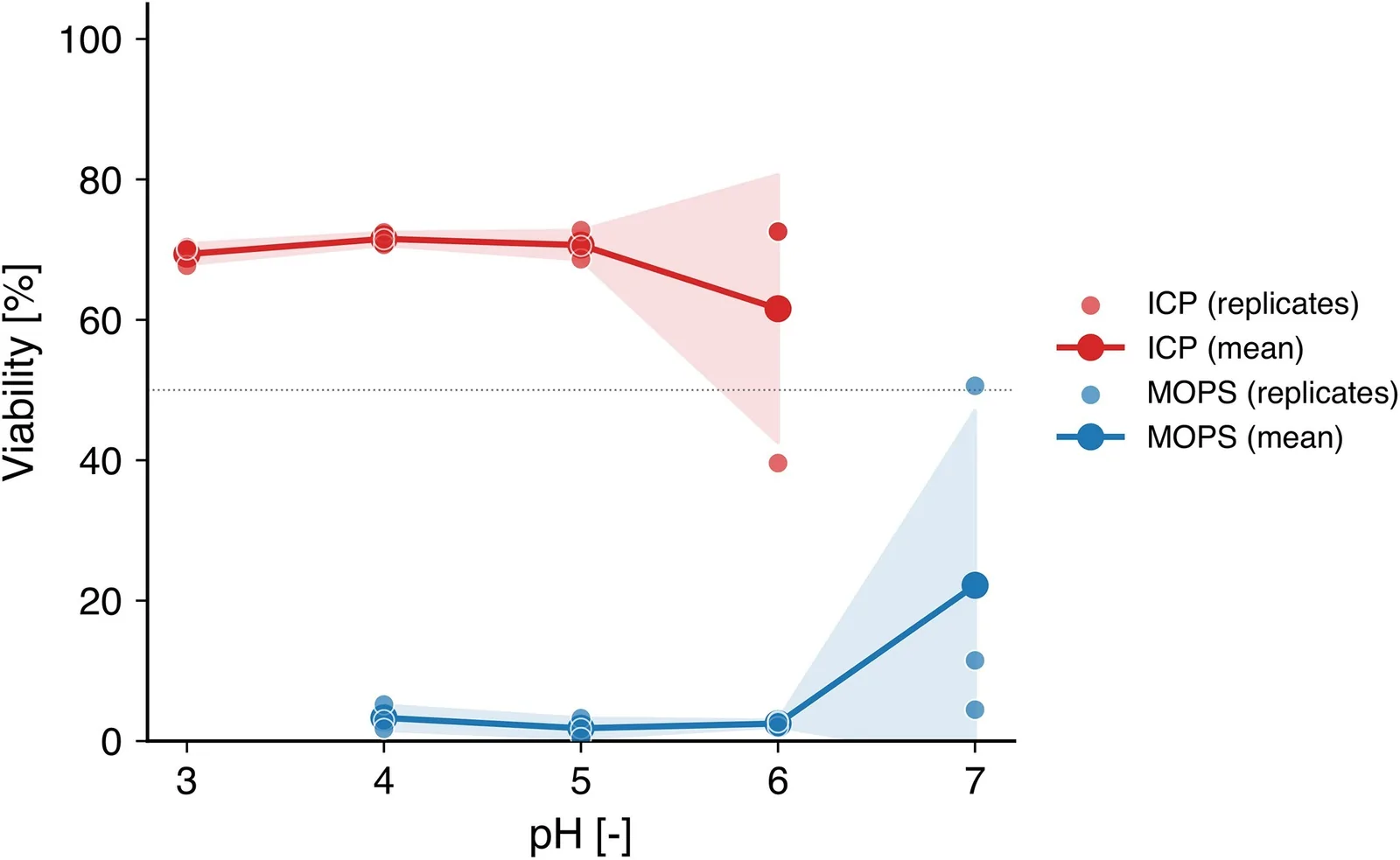
CFDA-based viability discrimination in *S. pastorianus* W-34/70 as a function of buffer composition and pH. Mean viable fraction ± SD across three biological replicates is shown for ICP and MOPS buffer across the tested pH range. ICP buffer at pH 3–5 provides stable and reproducible viability discrimination, whereas MOPS buffer yields unreliable estimates across all pH values tested.

CFDA produced the expected bimodal fluorescence distribution in the FITC channel, enabling discrimination of viable and nonviable subpopulations across all four strains. However, the optimal buffer and pH conditions for preserving both readouts simultaneously required systematic evaluation. In ICP buffer, stable and reproducible viability discrimination was achieved across pH 3–5 for *S. pastorianus* W-34/70, yielding mean viable fractions of 69.4 ± 1.4% (pH 3), 71.5 ± 0.9% (pH 4), and 70.6 ± 2.1% (pH 5) across biological triplicates (Fig. 2). At pH 6, interreplicate variability increased markedly (SD = 19.0%), indicating reduced robustness of viability discrimination under these conditions. In MOPS buffer, viability discrimination was strongly impaired across all tested pH values, with mean viable fractions below 4% at pH 4–6 and high interreplicate variability (SD up to 24.9% at pH 7), precluding reliable classification of viable and non-viable populations (Fig. 2). A pronounced elevation of PC7 autofluorescence was additionally observed in MOPS buffer at pH 4 and pH 5, requiring adjustment of the gating strategy to retain the full event population (Fig. S2). Based on these results, ICP buffer at pH 3–5 was selected as the standard condition for all subsequent combined age–viability measurements.

To confirm that mCherry–ChBD labeling does not interfere with CFDA-based viability discrimination, viability estimates from single CFDA staining were compared with those obtained under simultaneous double-staining conditions across pH 3–5. Both approaches yielded comparable population-level viability values, confirming that the presence of the mCherry–ChBD probe does not substantially alter CFDA uptake or retention (Fig. S4).

Viability thresholds for classifying individual cells as viable or nonviable were defined per strain group based on the bimodal valley of the log_10_(CFDA/PerCP) distribution at pH 3, where viable and nonviable populations are maximally separated (Fig. S1). A threshold ratio of 0.7 was applied to *S. pastorianus* W-34/70 and *K. phaffii* X33, and a threshold ratio of 10.0 was applied to *S. cerevisiae* var. *diastaticus* BE-134 and *S. cerevisiae* var. *chevalieri* LA-01. The ~10-fold difference in threshold values between the two groups likely reflects strain-specific differences in intracellular esterase activity, membrane permeability, and PerCP autofluorescence.

### GMM resolves discrete replicative age classes from mCherry–ChBD fluorescence

**Figure 3:**
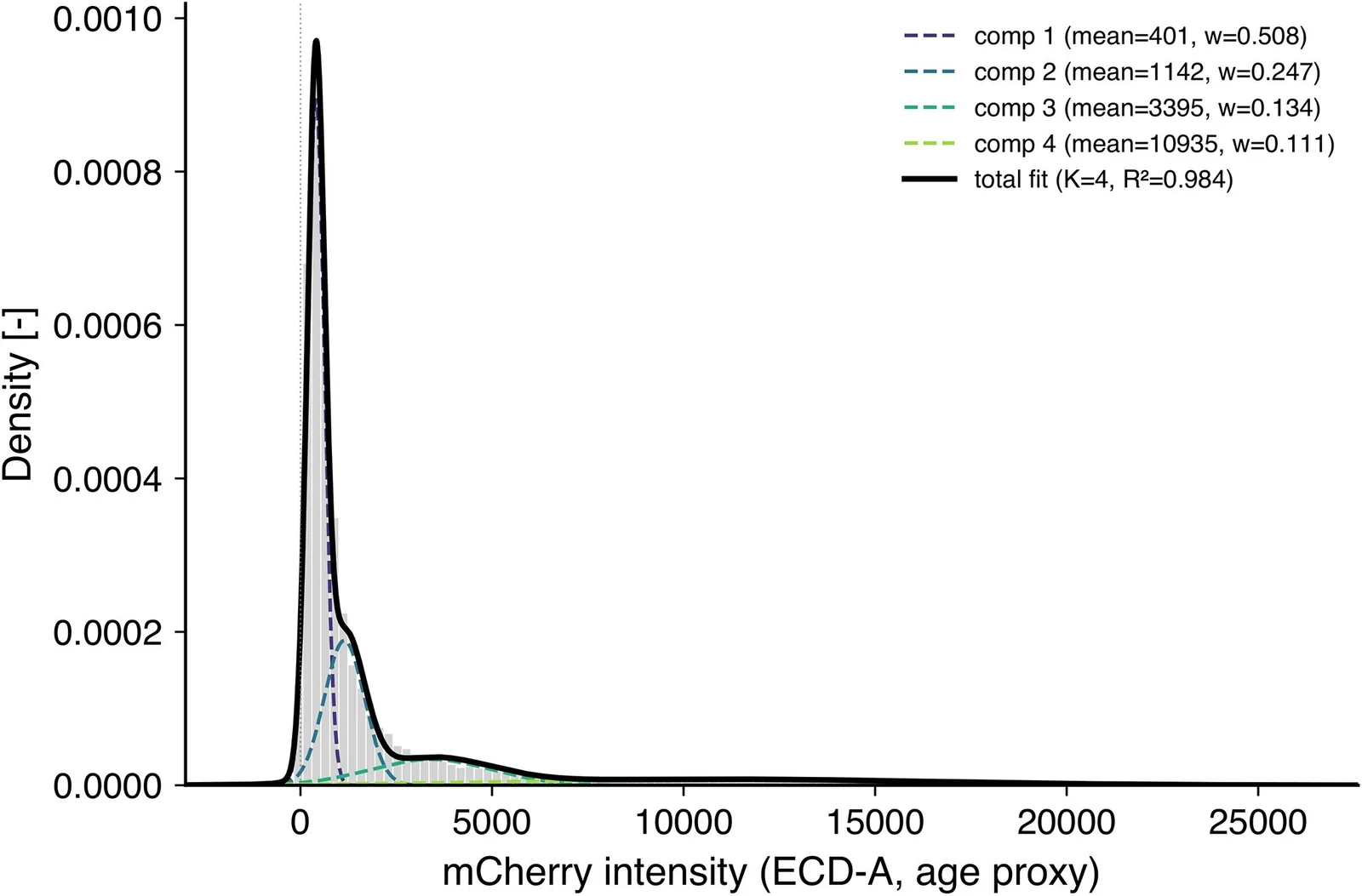
Representative Gaussian mixture model fit to mCherry–ChBD (ECD) fluorescence distributions in *S. pastorianus* W-34/70 under ICP buffer conditions at pH 3. The solid line represents the total model fit; dashed lines indicate individual Gaussian components corresponding to daughter cells (component 1, lowest fluorescence) and successive mother-cell generations (components 2–4).

**Figure 4:**
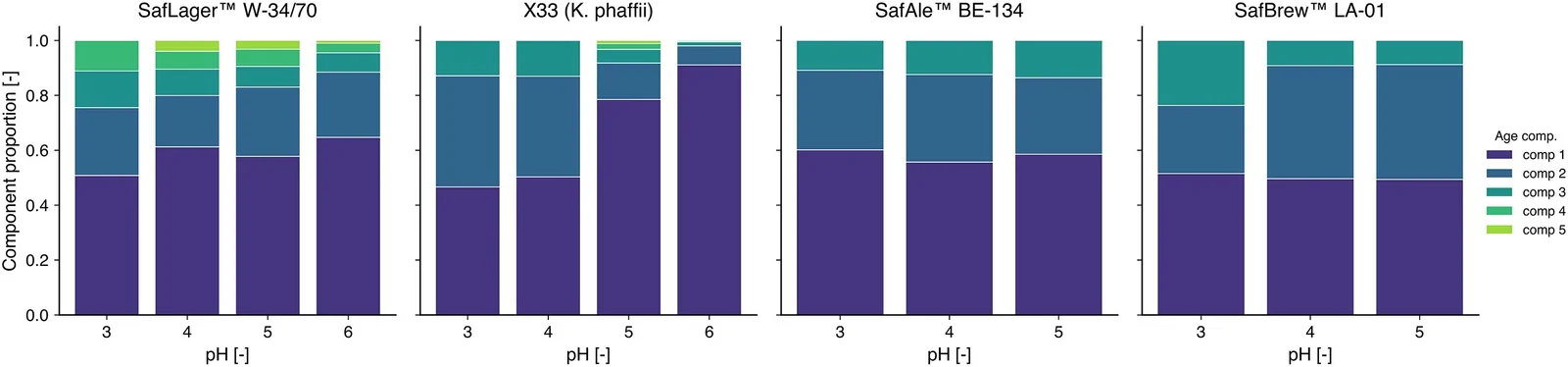
Age-class composition across pH conditions for all four yeast strains. Stacked bars show the relative contribution of each Gaussian mixture model component to the total population under ICP buffer conditions. Component 1 corresponds to daughter cells; higher components represent successive mother-cell generations with increasing replicative age.

Replicative age distributions were inferred from mCherry–ChBD fluorescence intensity in the ECD channel using GMM. Representative GMM fits resolved distinct fluorescence components corresponding to daughter cells and successive mother-cell generations across all four strains (Fig. 3). The dominant low-fluorescence component (component 1) consistently accounted for the largest fraction of the population, corresponding to the youngest cell class, with successive components shifted to higher ECD fluorescence values reflecting increasing bud-scar numbers. In *S. pastorianus* W-34/70, between three and five components were resolved under ICP buffer conditions, with the youngest age class (component 1) accounting for 58.6 ± 4.2% of the population and successive mother-cell generations each representing progressively smaller fractions (component 2: 23.1%; component 3: 9.4%; component 4: 6.8%; component 5: 2.7%). These frequencies closely track the geometric age distribution p(a) = 2^-(a+1)^ expected for an exponentially growing population under asymmetric division (50%, 25%, 12.5%, 6.25%, and 3.1% for the first five classes) (Hartwell and Unger 1977). The dominance of young cells and the rapid decline of older-cell frequencies are thus the expected demographic signature of a growing budding-yeast population rather than a novel biological feature; their close agreement with the independent GMM decomposition instead provides a parameter-free confirmation that the resolved components correspond to successive replicative-age classes. Population age composition remained stable across pH 3–5, indicating that neither buffer conditions nor CFDA staining distorted the intrinsic age structure of the population (Fig. 4).

Age class composition was similarly reproducible across biological replicates for all four strains, confirming that the GMM-based age stratification is robust under the experimental conditions applied. In MOPS buffer at pH 4–6, GMM fits yielded poor goodness-of-fit values (*R*^2^ < 0), consistent with the loss of age-associated fluorescence structure under these conditions, likely reflecting the predominance of non-viable cells with altered mCherry fluorescence. Age-structure analysis was therefore restricted to ICP buffer conditions for all strains.

### Forward scatter independently validates GMM-based age stratification and reveals strain-specific cell-size scaling

To independently validate the GMM-based age stratification, we examined whether cell size, approximated by forward scatter height (FSC-H), increases monotonically with assigned age component. Since FSC-H is not used as input to the GMM, a significant positive correlation between cell size and age component would provide independent biological support for the interpretation that GMM components capture genuine replicative age strata rather than staining artefacts.

**Figure 5:**
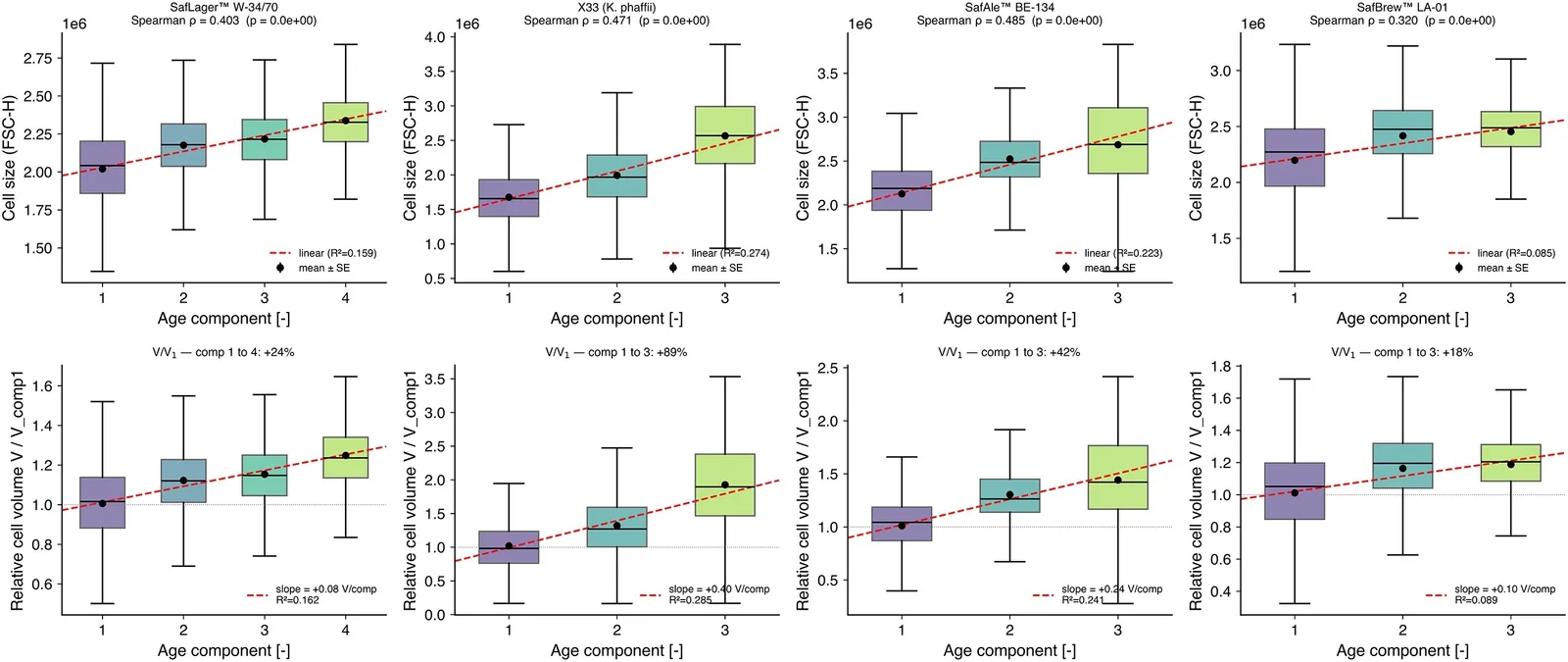
Cell-size scaling with replicative age class across all four yeast strains in ICP buffer at pH 3. **(Upper panels)** Forward scatter height (FSC-H) as a function of Gaussian mixture model component, shown with linear regression fit (R² given per panel) and Spearman rank correlation coefficient. **(Lower panels)** Relative cell volume per age component, calculated as V/V_1_ = (FSC/FSC_1_)^1.5^, illustrating the per-division volume increase for each strain. All correlations are statistically significant (*P* < 10^–15^), reflecting the large number of events analysed.

Spearman rank correlation revealed significant positive associations between FSC-H and GMM component in all four strains (*S. pastorianus* W-34/70: ρ = 0.40; *K. phaffii* X33: ρ = 0.47; *S. cerevisiae* var. *diastaticus* BE-134: ρ = 0.49; *S. cerevisiae* var. *chevalieri* LA-01: ρ = 0.32; all *P* < 10^–15^), confirming that mCherry–ChBD fluorescence intensity captures a biologically meaningful size-related age proxy across all strains (Fig. 5). Log-linear regression on *S. pastorianus* W-34/70 yielded a per-division cell volume increase of ~5.3%, broadly consistent in direction with the documented increase in cell size with replicative age in *S. cerevisiae*, where cell volume rises by ~150% over the first eight generations (Powell et al. 2003a). Notably, the per-division volume increase varied substantially across strains, ranging from 5.3% in *S. pastorianus* W-34/70 and 6.5% in LA-01 to 14.3% in BE-134 and 22.1% in *K. phaffii* X33, suggesting species- and strain-specific differences in cell-size regulation during replicative aging.

**Figure 6:**
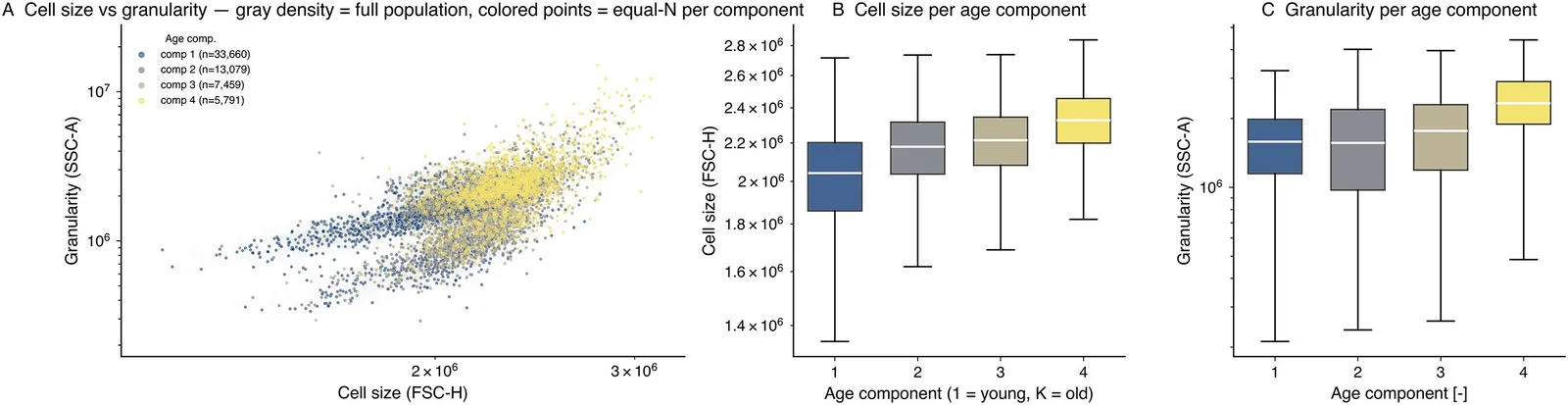
Joint FSC-H versus SSC-A scatter distribution of *S. pastorianus* W-34/70 colored by Gaussian mixture model age component. (A) Bivariate scatter with grey density overlay (total population) and colored component overlay (equal-n per component, Cividis color scale). Component 1 (youngest) occupies the low-FSC, low-SSC region; higher components shift progressively towards larger values. The continuous monomodal distribution is consistent with replicative aging as a physiological continuum, with the GMM components representing an operational stratification of that continuum. (B) FSC-H (linear scale) and (C) SSC-A (logarithmic scale) boxplots per age component, illustrating the increase in cell size and granularity with replicative age; the increase is monotonic for FSC-H, whereas SSC-A rises appreciably only from component 3 onwards.

Analysis of the joint FSC-H versus SSC-A scatter distributions, colored by GMM age component, revealed a continuous and monomodal population without discrete sub-clusters (Fig. 6). Component 1 (daughter cells) systematically occupied the low-FSC, low-SSC region of the scatter, while higher components were progressively shifted toward larger FSC-H and SSC-A values, consistent with the gradual accumulation of cell mass and internal complexity associated with replicative aging. A distinct low-SSC tail, comprising ~7% of all events, was dominated by component 1 cells (86% of low-SSC events), consistent with the presence of freshly budded daughter cells immediately after cytokinesis—small, low-granularity cells with minimal bud-scar accumulation. The continuous distribution along the FSC-H versus SSC-A diagonal further demonstrates that replicative aging in these yeast strains represents a gradual physiological continuum rather than a transition between discrete subpopulations.

Together, these results confirm that the GMM-based age stratification captures genuine replicative age classes, and that cell-size scaling with replicative age is a conserved but quantitatively strain-dependent feature of budding yeasts.

### Metabolic competence declines with replicative age in *S. pastorianus*

**Figure 7:**
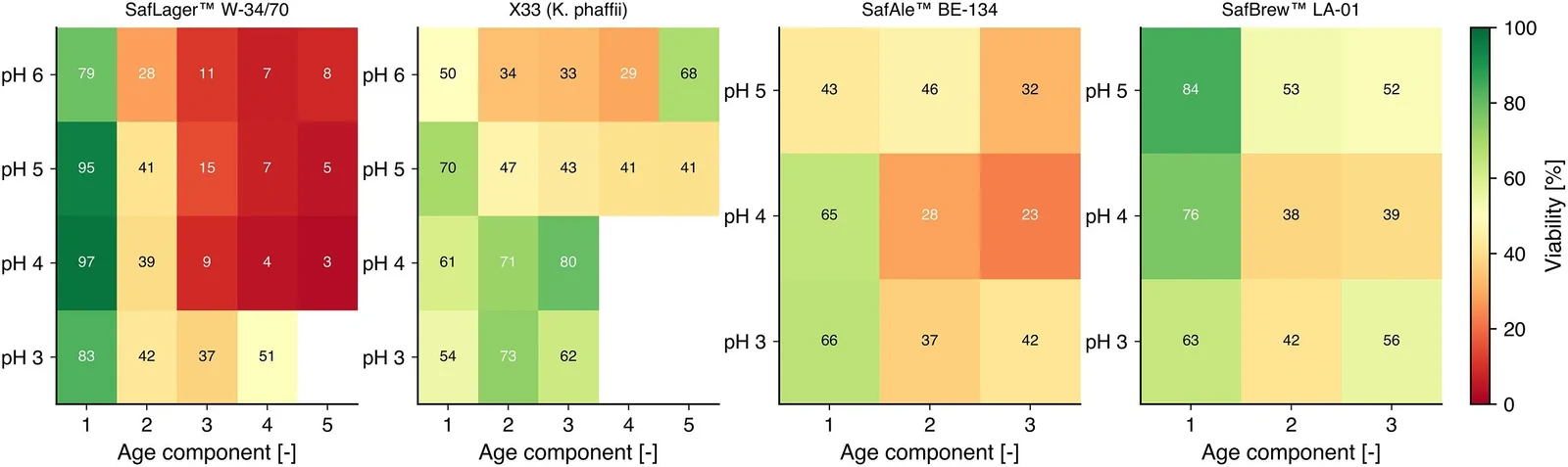
Age-class-specific metabolic viability in all four yeast strains under ICP buffer conditions, displayed as a heatmap. Each panel shows the mean viable fraction per Gaussian mixture model component (replicative age class) across pH 3–6 for S. pastorianus W-34/70 and K. phaffii X33, and across pH 3–5 for the two S. cerevisiae strains; blank cells indicate components not resolved under the respective condition. Component 1 corresponds to daughter cells; higher components represent successive mother-cell generations with increasing replicative age. Color intensity indicates viable fraction determined by CFDA/PerCP ratio classification.

To directly link replicative age with metabolic competence at single-cell resolution, component-specific viability was calculated by combining GMM-based age-class assignments with event-level CFDA/PerCP viability classification in *S. pastorianus* W-34/70. A pronounced decline in metabolic viability was observed across successive age classes under ICP buffer conditions (Fig. 7, Fig. S5). Daughter cells (component 1) exhibited the highest viability at 88%, whereas cells assigned to the first mother-cell generation (component 2) showed a substantially reduced viability of 38%. Older mother-cell populations displayed progressively lower viabilities, with mean values of 18% (component 3), 17% (component 4), and 5% (component 5), the latter representing the oldest and least abundant subpopulation (2.7% of the total population).

This age-dependent viability decline was reproducible across pH 4–6 in ICP buffer, confirming that the observed pattern reflects a genuine biological relationship between replicative age and metabolic competence rather than a pH-dependent measurement artefact. The steep drop in viability between component 1 (daughter cells, 88%) and component 2 (first mother-cell generation, 38%) is particularly striking, suggesting that even a single budding event is associated with a marked reduction in metabolic activity in this strain.

**Figure 8:**
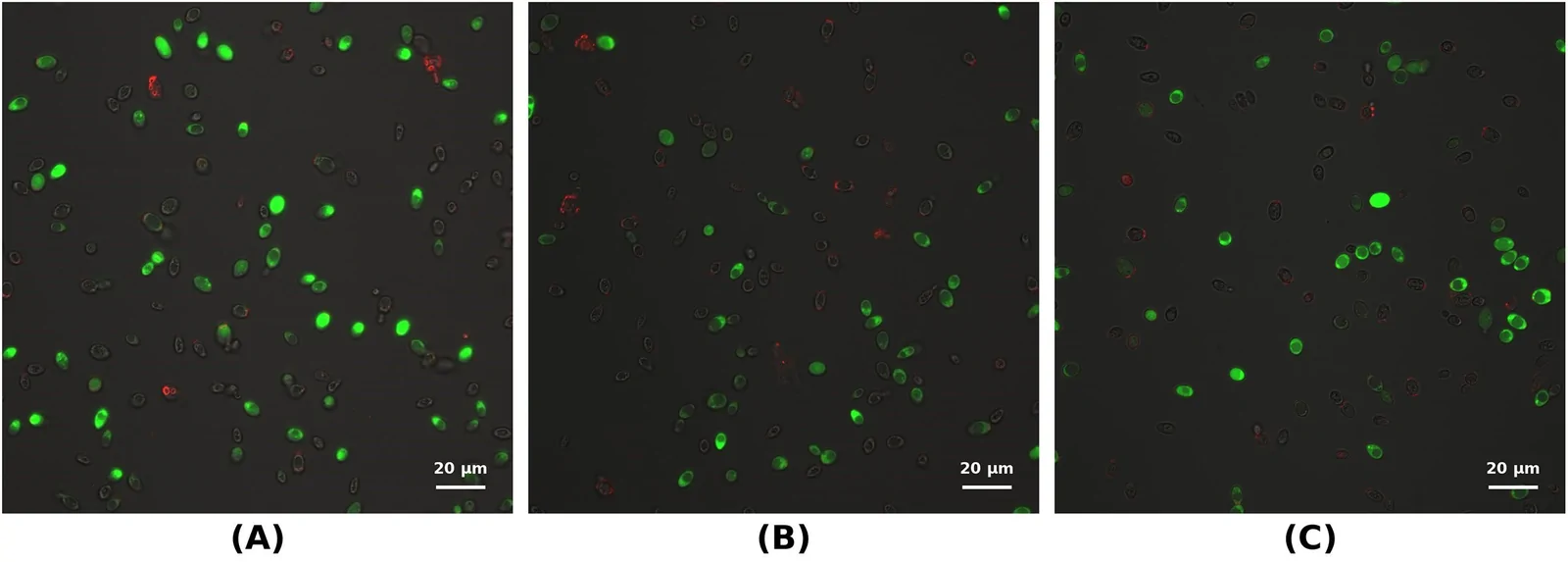
CLSM of S. pastorianus W-34/70 cells under simultaneous dual-parameter staining in ICP buffer at pH 4. (A–C) Three representative fields of view from one preparation. Green channel: CFDA-derived fluorescence in metabolically active cells with intact membranes. Red channel: mCherry fluorescence localized at bud-scar structures on the mother-cell surface. Gray channel: brightfield transmission overlay showing the total cell population. Cells showing both green and red fluorescence represent viable mother cells carrying at least one bud scar, directly demonstrating simultaneous and independent detection of metabolic viability and replicative age at single-cell resolution. Scale bar: 20 μm.

Simultaneous detection of mCherry–ChBD-labeled bud scars and CFDA-derived fluorescence was confirmed by CLSM in three representative fields of view under ICP buffer conditions at pH 4 (Fig. 8). Individual cells showing both red mCherry fluorescence at bud-scar structures and green CFDA-derived fluorescence confirm the independent and simultaneous readout of replicative age and metabolic viability within the same cell. Nonviable cells lacking CFDA fluorescence but retaining mCherry-labeled bud scars were also clearly distinguishable, demonstrating the discriminatory power of the dual-parameter approach at single-cell resolution. Equivalent confocal validation was performed for the two further *S. cerevisiae* strains examined in this study, *S. cerevisiae* var. *diastaticus* BE-134 (Fig. S6) and *S. cerevisiae* var. *chevalieri* LA-01 (Fig. S7); in both strains, colocalization of mCherry-labeled bud scars and CFDA fluorescence in mother cells, together with clearly distinguishable nonviable bud-scar-positive cells, reproduces the dual-parameter readout established for W-34/70 and confirms that the strategy transfers across metabolically distinct *S. cerevisiae* backgrounds.

### Age–viability coupling is strain-dependent among *S. cerevisiae* variants

To assess whether the pronounced age-dependent decline in metabolic competence observed in *S. pastorianus* W-34/70 is conserved across metabolically distinct *S. cerevisiae* strains, the combined age–viability workflow was applied to *S. cerevisiae* var. *diastaticus* BE-134 and *S. cerevisiae* var. *chevalieri* LA-01.

**Figure 9:**
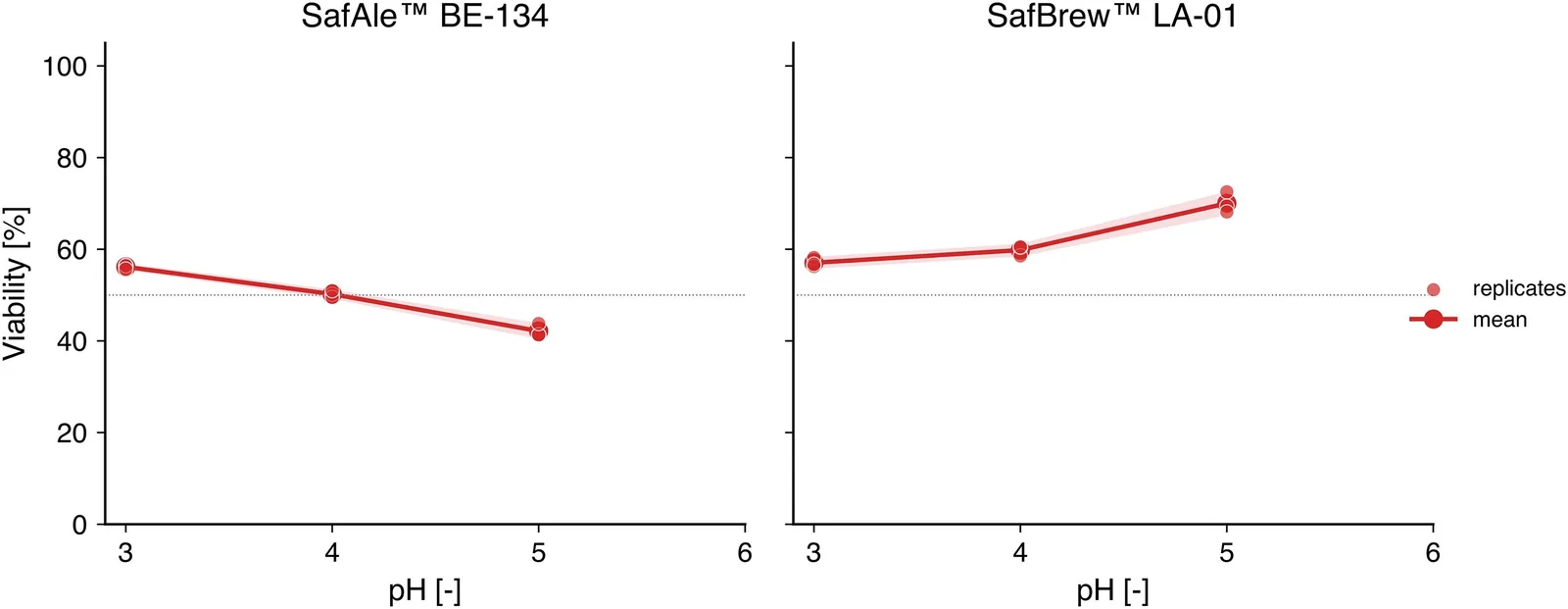
Population-level CFDA-based viability discrimination in *S. cerevisiae* var. *diastaticus* BE-134 and *S. cerevisiae* var. *chevalieri* LA-01 as a function of pH in ICP buffer. Mean viable fraction ± SD across three biological replicates is shown. BE-134 displays a declining viability trend with increasing pH, whereas LA-01 shows the opposite pattern, consistent with divergent acid-stress responses between the two strains.

Population-level viability measurements in ICP buffer revealed markedly different pH-dependent profiles between the two strains (Fig. 9). In BE-134, mean viable fractions decreased with increasing pH, from 56.2% at pH 3 to 50.2% at pH 4 and 42.2% at pH 5, indicating a higher sensitivity to reduced acid stress at elevated pH values. In contrast, LA-01 displayed the opposite trend, with viability increasing from 57.1% at pH 3 to 59.8% at pH 4 and 70.0% at pH 5, consistent with greater acid tolerance and improved metabolic activity under less acidic conditions. These divergent pH-dependent viability profiles reflect the distinct metabolic strategies of the two strains and underscore the importance of strain-specific assay optimization for CFDA-based viability measurements.

GMM-based age stratification resolved three discrete age classes in both BE-134 and LA-01 under ICP buffer conditions across pH 3–5. Component-specific viability analysis revealed detectable but variable age-dependent patterns in both strains (Fig. 7, Fig. S5). The age–viability relationship was less pronounced than observed for *S. pastorianus* W-34/70, with smaller differences in viable fraction between successive age classes. Nevertheless, a trend toward lower viability in older age classes was maintained in both strains, consistent with a general but strain-specific coupling between lineage history and metabolic competence across *S. cerevisiae* variants.

Independent validation by Spearman rank correlation between FSC-H and GMM component confirmed significant positive associations between cell size and replicative age in both strains (BE-134: ρ = 0.49, *P* < 10^–15^; LA-01: ρ = 0.32, *P* < 10^–15^), with per-division cell volume increases of ~14.3% and ~6.5%, respectively. The value observed for LA-01 is in close agreement with the 5.3% reported for *S. pastorianus* W-34/70, whereas BE-134 showed a somewhat higher per-division size increase, potentially reflecting differences in cell-cycle regulation associated with its hyperattenuating phenotype.

### Transfer to *K. phaffii* reveals a species-specific limitation of CFDA-based viability assessment

To evaluate the transferability of the dual-parameter workflow to a non-*Saccharomyces* host, the combined age–viability analysis was applied to *K. phaffii* X33. Bud-scar labeling with the mCherry–ChBD fusion protein produced a clear and reproducible increase in ECD fluorescence relative to unstained control cells, confirming effective surface binding and detection of age-associated fluorescence in *K. phaffii*. Fluorescence in the FITC channel remained unchanged in the absence of CFDA, indicating minimal spectral bleed-through and preservation of channel independence.

Population-level CFDA-based viability measurements in ICP buffer yielded stable and reproducible mean estimates across pH 3–5, with mean viable fractions of 62.8 ± 0.7% (pH 3), 66.2 ± 0.6% (pH 4), and 65.8 ± 0.7% (pH 5) (Fig. S3). At pH 6, interreplicate variability increased markedly (SD = 6.2%), consistent with reduced discriminatory power of CFDA staining under less acidic conditions, as observed for *S. pastorianus* W-34/70. In MOPS buffer, viability estimates were highly variable and unreliable across all tested pH values (SD up to 25.8% at pH 4), precluding meaningful interpretation, in agreement with the findings for *S. pastorianus* W-34/70.

Importantly, CFDA-based viability discrimination in *K. phaffii* X33 was found to be dependent on the growth phase of the culture. Reliable bimodal separation of viable and nonviable populations was obtained exclusively from overnight cultures in active growth (OD_600_ = 4.5 ± 0.3), as confirmed by continued biomass accumulation throughout the cultivation period. Measurements performed on cultures that had reached stationary phase yielded unreliable discrimination between viable and nonviable populations and were excluded from further analysis. This growth-phase dependency of CFDA uptake in *K. phaffii* has not been previously reported and represents an important methodological boundary for the application of CFDA-based viability assays in this host.

GMM-based age stratification resolved three discrete age classes in *K. phaffii* X33 under ICP buffer conditions at pH 3 and pH 4 (Fig. 4), and five classes at pH 5. Component-specific viability analysis revealed strongly pH-dependent patterns that diverge markedly from those observed in *Saccharomyces* strains (Fig. 7, Fig. S5). At pH 3 and pH 4, a statistically significant *positive* association between replicative age and CFDA-derived viability was observed (pH 4: ρ = +0.949, *P* < .001), inconsistent with established models of replicative aging. At pH 5, the expected negative association was recovered (ρ = −0.666, *P* = .007), consistent with an age-dependent decline in metabolic competence, though interreplicate variability remained elevated.

These opposing trends are unlikely to reflect genuine biological differences and more plausibly arise from methodological artefacts specific to *K. phaffii* under acidic ICP buffer conditions. First, *K. phaffii* undergoes pronounced pH-dependent flocculation at pH 4.0, mediated by differential expression of flocculin genes, which is fully reversible at higher pH values (De et al. 2025). Cell aggregation under these conditions may confound single-cell cytometric measurements by altering the apparent fluorescence of age-class subpopulations. Second, CFDA uptake in yeast is limited primarily by transmembrane transport rather than intracellular esterase activity (Breeuwer et al. 1995), and the distinct cell wall architecture of *K. phaffii* may render CFDA permeability more sensitive to external pH in a cell-age-dependent manner. Third, the standard operating pH range for *K. phaffii* bioprocesses is pH 5.0–6.5 (De et al. 2025), indicating that ICP buffer at pH 3–4 represents an atypical stress condition for this species. Taken together, these findings indicate that the ICP buffer conditions established for *Saccharomyces* strains are not directly transferable to *K. phaffii*, and that CFDA-based viability discrimination in this species requires dedicated buffer optimisation at pH values closer to its physiological optimum.

Independent validation by Spearman rank correlation between FSC-H and GMM component confirmed a significant positive association between cell size and replicative age in *K. phaffii* X33 (ρ = 0.47, *P* < 10^–15^), with a per-division cell volume increase of ~22.1%, substantially higher than the 5.3% observed for *S. pastorianus* W-34/70. This difference may reflect species-specific differences in cell-size regulation during replicative aging.

Together, these results demonstrate that the mCherry–ChBD-based dual-parameter strategy is transferable to *K. phaffii*, but also reveal that reliable CFDA-based age-class viability discrimination in *K. phaffii* is critically dependent on buffer pH, and that the ICP buffer conditions optimised for *Saccharomyces* strains are not directly applicable to this species without further method adaptation. The growth-phase dependency of CFDA uptake further highlights the importance of validating fluorescence-based viability assays under species- and condition-specific experimental parameters before drawing biological conclusions.

### Lineage-resolved process monitoring reveals progressive viability decline in aged subpopulations during fermentation

**Figure 10:**
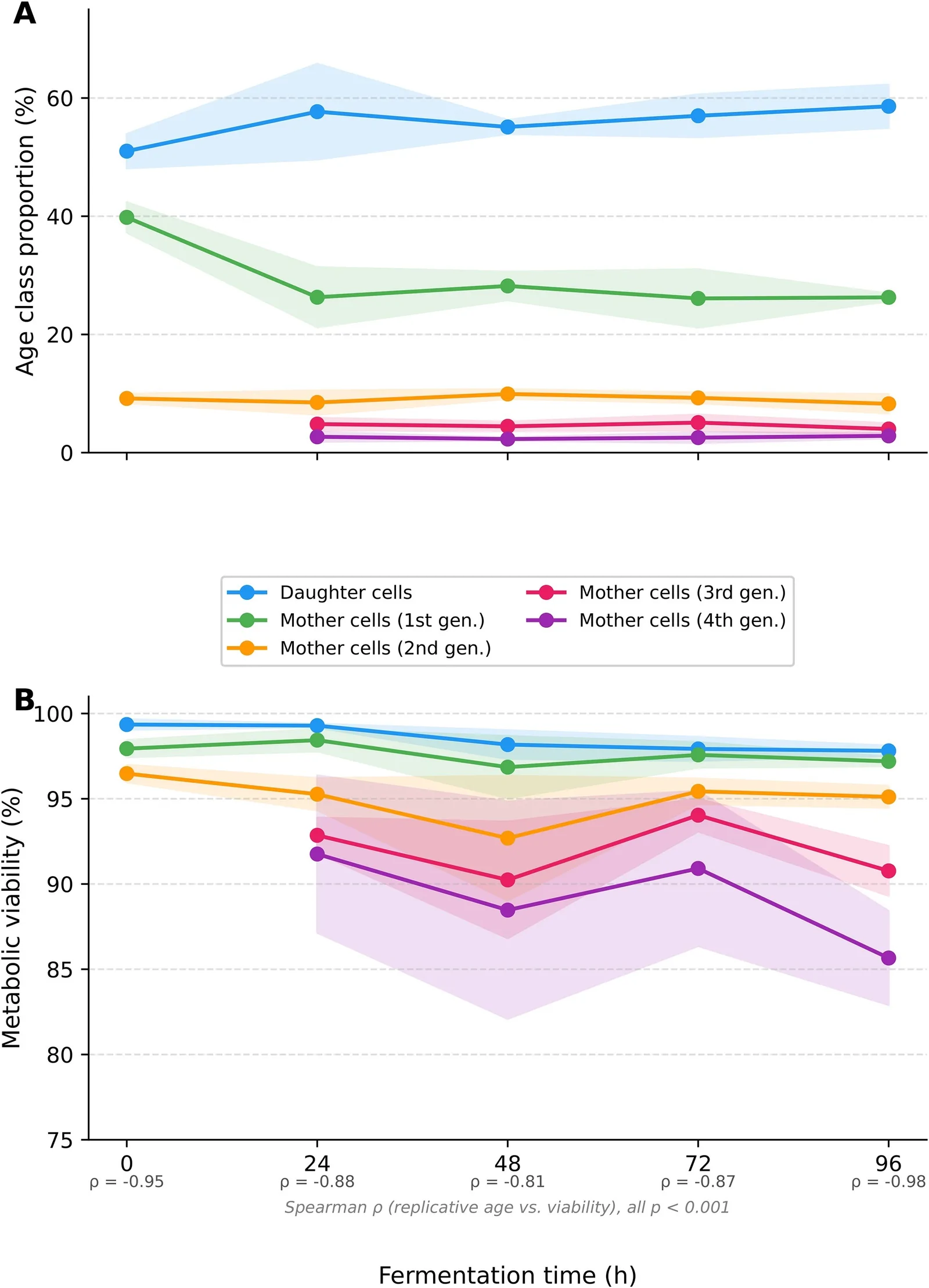
Lineage-resolved fermentation monitoring of *S. pastorianus* W-34/70 over 96 h. (A) Age class proportion over fermentation time. Stacked line plots show the mean relative contribution of each Gaussian mixture model component (replicative age class) to the total population across five fermentation time points (0, 24, 48, 72, and 96 h). Shaded areas represent ± SD across three independent biological replicates. (B) Age-class-specific metabolic viability over fermentation time. Mean viable fraction per age class is shown as a function of fermentation time. Shaded areas represent ± SD across three biological replicates. Spearman rank correlation coefficients (ρ) between replicative age class and metabolic viability are indicated below the *x*-axis for each time point; All measurements were performed under ICP buffer conditions at pH 4.

To evaluate whether the dual-parameter framework can capture dynamic changes in population age structure and metabolic competence over the course of an industrial fermentation, *S. pastorianus* W-34/70 was monitored at five time points spanning a 96 h cultivation (0, 24, 48, 72, and 96 h). At each time point, three independent biological replicates were subjected to simultaneous mCherry–ChBD bud-scar labeling and CFDA-based viability staining under ICP buffer conditions at pH 4, and GMM-based age stratification was applied to each sample individually (Fig. 10).

At the inoculation time point (*t* = 0 h), three discrete age classes were resolved, with daughter cells (component 1) accounting for 51.0 ± 2.9% of the population. From *t* = 24 h onward, GMM decomposition consistently resolved five age classes across all biological replicates, reflecting the progressive accumulation of older cell subpopulations as the fermentation proceeded. The proportion of daughter cells remained stable throughout the cultivation, ranging from 55.1 ± 1.2% at 48 h to 58.6 ± 3.7% at 96 h, indicating continuous population renewal over the fermentation period (Fig. 10A).

Age-class-specific viability measurements revealed a monotonically declining gradient from daughter cells to the oldest mother-cell subpopulation at every time point examined (Fig. 10B). Spearman rank correlation between replicative age class and metabolic viability was statistically significant at all five time points (*t* = 0 h: ρ = −0.949; *t* = 24 h: ρ = −0.884; *t* = 48 h: ρ = −0.807; *t* = 72 h: ρ = −0.873; *t* = 96 h: ρ = −0.982; all *P* < .001), and the trend was monotonically decreasing at each time point. Daughter cells maintained high metabolic viability throughout the cultivation (99.4 ± 0.3% at 0 h; 97.8 ± 0.3% at 96 h), whereas the oldest subpopulation (component 5) showed a progressive decline, reaching 85.7 ± 2.8% at 96 h. The viability gradient between daughter cells and the oldest mother-cell subpopulation widened over time, from 7.5 percentage points at 24 h to 12.1 percentage points at 96 h, consistent with a progressive accumulation of physiological damage in older cell lineages over the course of the fermentation.

Weighted population-level viability remained high and relatively stable throughout the cultivation (98.5 ± 0.3% at 0 h; 96.8 ± 0.3% at 96 h), demonstrating that population-level viability estimates alone do not capture the age-dependent heterogeneity resolved at single-cell resolution by the dual-parameter approach. Together, these results demonstrate that the lineage-aware framework presented here is applicable to longitudinal process monitoring and can resolve age-dependent physiological trajectories that are masked by conventional population-level measurements.

## Discussion

The present study establishes a dual-parameter single-cell framework that directly links replicative age with metabolic competence across a panel of industrially relevant yeast strains. By relocating the bud-scar readout from the green to the red spectral channel using an mCherry–ChBD fusion protein (Eigenfeld et al. 2021a), we enabled simultaneous flow-cytometric quantification of lineage history and physiological state within individual cells without spectral interference or computational autofluorescence correction. This design overcomes a fundamental limitation of established GFP- or FITC-based age markers, which spectrally overlap with CFDA-based viability readouts and with intrinsic yeast autofluorescence (Maslanka et al. 2018, Eigenfeld et al. 2022). The strict spectral orthogonality between mCherry-based age labeling and CFDA-derived viability signals, confirmed both by flow cytometry and CLSM, provides a technically straightforward platform for multiplexed single-cell phenotyping without model-based correction.

A central observation enabled by this workflow is the pronounced decline in metabolic competence with increasing replicative age observed in *S. pastorianus* W-34/70 and other *Saccharomyces* strains. Daughter cells displayed the highest viability (88% in W-34/70), whereas successive mother-cell generations exhibited progressively reduced metabolic activity, with the oldest resolved class (component 5, corresponding to only about five divisions and ∼3% of the population) retaining only 5% viability. The steep drop between daughter cells and first-generation mother cells (88%–38%) is particularly striking, suggesting that even a single budding event is already associated with a marked reduction in CFDA-defined metabolic competence in this strain. This pattern is consistent with established models of replicative aging in budding yeasts, which describe age-associated impairments in mitochondrial function, reduced stress tolerance, and declining growth capacity (Leupold et al. 2019, Yang et al. 2022). Replicative aging has further been linked to asymmetric segregation of damaged cellular components between mother and daughter cells, resulting in rejuvenation of newly formed daughters and progressive functional decline in older mothers (Henderson et al. 2014, Knorre et al. 2018). The present data show that, for the intended application, lineage history can be read out as a strong single-cell predictor of physiological competence rather than only as a population average.

The divergent pH-dependent CFDA fluorescence profiles observed between *S. cerevisiae* var. *diastaticus* BE-134 and *S. cerevisiae* var. *chevalieri* LA-01 highlight the strong strain dependency of CFDA-based readouts. BE-134 exhibited decreasing fluorescence intensity with increasing pH, whereas LA-01 showed the opposite trend. These differences are unlikely to directly reflect changes in cell viability, but rather indicate strain-specific variation in CFDA uptake, intracellular esterase activity, fluorescence retention, and/or efflux processes, all of which are known to be pH-sensitive (Diaper and Edwards 1994, Breeuwer et al. 1995).

The opposing response patterns emphasize that CFDA-derived signals cannot be interpreted as direct proxies for viability without prior assay calibration. Instead, strain-specific optimization and threshold definition are required to account for differences in dye processing and intracellular physiology. The ~10-fold variation in CFDA/PerCP threshold values between the two strain groups further supports the presence of substantial biological differences affecting dye conversion and retention, rather than uniform shifts in viability.

The age–viability coupling observed in BE-134 and LA-01 was weaker and less pronounced than in *S. pastorianus* W-34/70, with smaller differences in viable fraction between successive age classes. This observation suggests that the relationship between lineage history and physiological state is present across strains but not uniformly conserved in magnitude. Potential contributing factors include differences in the efficiency of asymmetric damage segregation, the rate of mitochondrial dysfunction accumulation, and the capacity for stress adaptation—all of which are known to vary across *S. cerevisiae* strains (Eigenfeld et al. 2021b, Knorre et al. 2018). The comparative framework established here provides a practical approach to systematically quantify these differences across strains of industrial relevance.

Application of the same workflow to *K. phaffii* X33 demonstrated that the mCherry–ChBD-based dual-parameter strategy is transferable to non-*Saccharomyces* hosts, but also revealed a markedly weaker coupling between replicative age and metabolic competence in this species. Although replicative age subpopulations could be resolved by GMM, age-dependent declines in viability were less pronounced and more uniformly distributed across age classes. This observation is consistent with the distinct cell wall architecture, metabolic regulation, and stress-response strategies of *K. phaffii* compared to conventional *Saccharomyces* hosts (Gangl et al. 2025, Cheng et al. 2025), and suggests that the physiological consequences of replicative aging are species-specific rather than universally conserved. These findings highlight the importance of comparative single-cell analyses across species to avoid overgeneralizing aging phenotypes derived primarily from *Saccharomyces* model systems.

An important methodological finding of this study is the growth-phase dependency of CFDA uptake in *K. phaffii* X33. Reliable bimodal viability discrimination was achieved exclusively in cultures confirmed to be in active exponential growth, whereas stationary-phase cultures yielded unreliable signal separation. This observation, which has not been previously reported for *K. phaffii*, likely reflects growth-phase-dependent changes in membrane permeability or esterase activity that affect CFDA uptake and retention (Weigert et al. 2009). This effect may be attributed to growth-phase-dependent physiological changes, including reduced metabolic activity, altered membrane composition, and impaired intracellular pH homeostasis in stationary-phase cells, all of which can influence CFDA uptake, hydrolysis, and fluorescence retention (Breeuwer et al. 1995). While *K. phaffii* exhibits distinct metabolic adaptations depending on the available carbon source, the observed effect is more likely driven by general growth-phase-dependent changes rather than methylotrophy per se. Previous studies have demonstrated that mCherry exhibits high stability across acidic pH ranges (Shaner et al. 2004, Botman et al. 2019), supporting its suitability for assays that rely on CFDA-based readouts under low pH conditions. The observed loss of CFDA bimodality at higher pH values and in stationary phase underscores that multiparameter cytometric measurements are constrained not only by spectral compatibility but also by physicochemical factors that influence probe chemistry and cellular physiology. Systematic validation of assay conditions is therefore essential when applying CFDA-based viability measurements to nonconventional yeast hosts. Across all strains tested, pH 4 in ICP buffer yielded consistent CFDA fluorescence profiles that allowed reliable discrimination of cell populations. In parallel, the fluorescence signal of the mCherry reporter remained stable under these conditions.

Independent validation of the GMM-based age stratification by FSC-H correlation confirmed that mCherry–ChBD fluorescence components capture genuine replicative age strata across all four strains. The per-division cell volume increase of ~5.3% observed for *S. pastorianus* W-34/70 is of the same order as the cell-volume increase documented for *S. cerevisiae* over the replicative lifespan (~150% across the first eight generations) (Powell et al. 2003a), providing biological support for the GMM decomposition. The substantially higher per-division volume increases observed for *K. phaffii* X33 (22.1%) and BE-134 (14.3%) suggest species- and strain-specific differences in cell-size regulation during replicative aging that warrant further investigation. The continuous monomodal FSC-H versus SSC-A scatter distribution, with component 1 dominating the low-SSC tail, further confirms that replicative aging in budding yeasts represents a physiological continuum rather than a transition between discrete subpopulations—a finding consistent with the gradual nature of age-associated cellular changes described in the literature (Sorokin et al. 2014, Knorre et al. 2018).

An intrinsic feature of any bud-scar-based cytometric assay is that it resolves the demographically dominant, early-to-moderate portion of the replicative lifespan rather than its terminal, senescent stages. In an exponentially growing, randomly sampled population, the fraction of cells that have completed a divisions follows the geometric relationship p(a) = 2^-(a+1)^, which arises directly from asymmetric division (Hartwell and Unger 1977). Cells carrying ~20 bud scars, close to the mean replicative lifespan of *S. cerevisiae* of 20–25 generations (Mortimer and Johnston 1959), therefore occur at a frequency of roughly 5 × 10^–7^, corresponding to fewer than one cell per 10^6^ recorded events and effectively none within a typical 20 000-event gate. The resolution of approximately five age classes by the GMM is thus not an arbitrary cut-off but a direct consequence of population demographics: these classes already encompass ~97% of the population, whereas classically senescent cells are demographically negligible in a freely growing culture. This is precisely why dedicated enrichment strategies, including biotin–streptavidin magnetic sorting, mother-enrichment approaches, and microfluidic mother-cell tracking, were developed specifically to access late-life cells (Eigenfeld et al. 2023b, Crane et al. 2014, Jo et al. 2015). The workflow presented here is therefore positioned to characterise the process-relevant majority of a fermenting population rather than to probe terminal senescence, and its age-resolved viability readout should be interpreted in that light.

Buffer composition and pH were identified as critical determinants for preserving both age-associated fluorescence structure and viability discrimination. ICP buffer at pH 3–4 provided the most reliable conditions across all strains, whereas MOPS buffer strongly impaired CFDA-based viability discrimination, likely due to its interference with CFDA uptake or intracellular retention. The pronounced elevation of PC7 autofluorescence observed in MOPS buffer at pH 4–5 represents a previously unreported phenomenon that required adaptation of the gating strategy to retain the full event population. This effect, which was restricted to MOPS buffer under acidic conditions and absent in ICP buffer, may reflect a pH-dependent interaction between the MOPS zwitterion and yeast cell wall components—a finding that should be considered when designing flow cytometric assays under acidic conditions.

The longitudinal fermentation monitoring over 96 h further demonstrates the practical utility of lineage-aware single-cell analysis for industrial bioprocesses. While weighted population-level viability remained high and stable throughout the cultivation (98.5% at 0 h to 96.8% at 96 h), the age-resolved analysis revealed a progressive widening of the viability gradient between daughter cells and the oldest mother-cell subpopulation, from 7.5 percentage points at 24 h to 12.1 percentage points at 96 h. This divergence between aggregate and lineage-resolved measurements illustrates that conventional population-level viability assessments systematically underestimate the heterogeneity present in aging fermentation populations and may obscure early indicators of process decline. Lineage-resolved monitoring may therefore serve as a sensitive readout for detecting age-associated physiological deterioration before it manifests at the population level, with potential applications in process control, strain evaluation, and the design of cultivation strategies that mitigate accumulation of aged subpopulations during long-term fermentations (Eigenfeld et al. 2023b, Eigenfeld and Schwaminger 2025).

Several limitations of the present study should be considered. First, the method relies on surface labeling of chitin-rich bud scars and is therefore restricted to organisms that undergo asymmetric division with persistent scar structures. Second, GMM-based age classification assumes discrete generational subpopulations and may become less reliable when age distributions are highly compressed or when fluorescence resolution is reduced, as observed under MOPS buffer conditions. Third, the CFDA-based viability readout reflects a composite of esterase activity and membrane integrity, and may not capture all dimensions of cellular metabolic competence. Future work should therefore explore alternative viability indicators, extended validation under process-relevant conditions, and integration with real-time or online flow cytometric monitoring strategies.

Fourth, all strains were cultivated under identical conditions at 30°C to ensure comparability across species; however, this temperature exceeds the typical propagation range for lager strains such as *S. pastorianus* W-34/70 (ordinarily ≤ 27°C) and may have introduced strain-specific thermal stress. Future studies should assess whether comparable age–viability gradients are reproduced under strain-optimised cultivation temperatures.

Despite these limitations, the present study establishes a generalizable framework for dual-parameter single-cell phenotyping that directly links lineage history with physiological state across a diverse panel of industrially relevant yeast strains. By enabling age-resolved viability measurements without spectral interference, this approach provides new opportunities to investigate aging dynamics, population heterogeneity, and fitness trade-offs in yeast systems relevant to biotechnology and fermentation science. In applied contexts, lineage-aware analysis may support improved strain evaluation, optimization of cultivation strategies, and the development of interventions aimed at mitigating age-associated performance decline during long-term cultivations.

## Conclusion

This study establishes a red-channel bud-scar labeling strategy that enables simultaneous flow-cytometric quantification of replicative age and metabolic viability in individual yeast cells. By relocating the age-dependent fluorescence readout from the green to the red spectral channel using an His_6_–SUMO–mCherry–ChBD fusion protein, the method eliminates spectral interference with CFDA-based vitality assays and allows direct multiparameter single-cell phenotyping without computational autofluorescence correction. Independent validation by forward scatter correlation confirmed that GMM-based age components capture genuine replicative age strata across all four strains, with per-division cell volume increases consistent with published estimates for budding yeasts.

Application of this framework to a panel of four industrially relevant yeast strains revealed that the coupling between replicative age and metabolic competence is strongly strain- and species-dependent. In *S. pastorianus* W-34/70, metabolic viability declined monotonically from 88% in daughter cells to 5% in the oldest resolved mother-cell class, establishing a clear age-resolved link between lineage history and metabolic competence. Among *S. cerevisiae* variants, BE-134 and LA-01 displayed divergent pH-dependent viability profiles reflecting their distinct metabolic strategies, with weaker but detectable age-dependent viability gradients in both strains. Transfer to *K. phaffii* X33 demonstrated the generalizability of the approach to non-*Saccharomyces* hosts, while simultaneously revealing a growth-phase dependency of CFDA-based viability discrimination in this species that has not been previously reported.

Together, these findings demonstrate that replicative age is a strain-dependent rather than universally conserved determinant of cellular performance, and that its impact cannot be generalized across yeast species. The dual-parameter workflow introduced here translates this insight into a practical, near-routine tool for industrial settings. As a concrete example, a brewery quality-control laboratory could apply the assay to a pitching yeast slurry before each fermentation: a single 30-min staining and flow cytometry run resolves both the proportion of aged mother-cell generations and their individual viability, enabling an evidence-based decision on whether the slurry is suitable for repitching or should be replaced with a fresh propagation. It should be emphasised, however, that age-resolved viability data constitute one component within a broader quality-control framework; evidence-based pitching decisions also require concurrent assessment of microbiological purity, absence of contaminating organisms, and other fermentation-relevant parameters. In this context, and depending on the harvesting regime, replicative age can become an additional risk factor alongside microbiological contamination and genetic drift: under cropping practices that preferentially retain larger, older, more flocculent cells, successive repropagation may progressively enrich the population for aged mother-cell generations, whereas near-random harvesting largely preserves the age structure and need not accumulate aged cells (Powell et al. 2003b, Bühligen et al. 2014). Monitoring the age structure across propagation cycles therefore offers a rational, process-specific basis for deciding when a pitching yeast should be replaced. In the longitudinal monitoring presented here, this readout detected a progressive widening of the daughter-to-mother-cell viability gradient (from 7.5 to 12.1 percentage points over 96 h) that remained completely masked by the population-level viability of ≥ 96.8 % throughout the cultivation, an early-warning signature for fermentation performance decline that conventional assays cannot deliver. Beyond brewing, the same approach allows side-by-side, age-resolved comparison of candidate strains during bioprocess development, identifying which strains accumulate aged, low-viability subpopulations earliest under defined cultivation conditions. Because of the spectral orthogonality of the mCherry–ChBD age probe, additional functional readouts (intracellular pH, reactive oxygen species, or enzyme activity) can be integrated into the same cytometric panel without computational unmixing, extending the framework toward comprehensive single-cell metabolic fingerprinting of industrial yeast populations.

## Supplementary Material

foag037_Supplemental_File

## Data Availability

Raw flow cytometry data files (FCS format) are publicly available on Zenodo as four strain-specific datasets: *S. pastorianus* W-34/70 (https://doi.org/10.5281/zenodo.20157503), *S. cerevisiae* var. *diastaticus* BE-134 (https://doi.org/10.5281/zenodo.20157855), *S. cerevisiae* var. *chevalieri* LA-01 (https://doi.org/10.5281/zenodo.20157924), and *K. phaffii* X33 (https://doi.org/10.5281/zenodo.20157668). Additional analysis scripts are available from the corresponding author upon reasonable request.
