# Supplementary material for: Replicative age differentially predicts metabolic competence across industrial yeasts at single-cell resolution
Source: FEMS Yeast Res. 2026 Aug 7;26:foag037. doi: 10.1093/femsyr/foag037 (PMC13499230; doi:10.1093/femsyr/foag037)
Supplement: Supplementary file 1 [file foag037_supplemental_file.docx]

**Supplementary Information**

Replicative age differentially predicts metabolic competence across industrial yeasts at single-cell resolution

Marco Eigenfeld, Elisabeth Zach, Ann-Kathrin Bürkle, Benjamin Schneider, Sebastian P. Schwaminger

This file contains Supplementary Tables S1–S4 and Supplementary Figures S1–S7.

**Supplementary Tables**

**Table S1.** Primer sequences used for Gibson assembly of the His₆–SUMO–mCherry–ChBD expression construct. All primers were synthesized by Invitrogen (Thermo Fisher Scientific; Order No. 669553 01). Sequences are given 5'–3'.

| **ID** | **Name** | **Sequence (5'–3')** | **Function** |
| --- | --- | --- | --- |
| F05 | Backbone_fwd_mCherry | GGT GGC GGT GGT AGC GGT G | Linearize pET28b(+) backbone |
| F06 | Backbone_rev_mCherry | GGA CCC ACC GCC ACC GGA | Linearize pET28b(+) backbone |
| F07 | RFP_fwd | GGT CCG GTG GCG GTG GGT CCG TGA GCA AGG CGG AGG AG | Amplify mCherry; overlaps vector upstream |
| F08 | RFP_rev | CCA CCG CTA CCA CCG CCA CCC TTG TAC AGC TCG TCC ATG C | Amplify mCherry; overlaps ChBD downstream |

**Table S2.** Compositions of lysis, IMAC binding, and IMAC elution buffers used for purification of the His₆–SUMO–mCherry–ChBD fusion protein. Lysis/wash buffer adjusted to pH 7.3; binding and elution buffers adjusted to pH 7.5 with NaOH or HCl as required. All amounts per final volume indicated.

| **Component** | **Lysis/Wash buffer** | **Binding buffer** | **Elution buffer** | **EDTA stock** |
| --- | --- | --- | --- | --- |
| NaCl | 8.766 g | 29.22 g | 29.22 g | – |
| MOPS | 5.23 g | – | – | – |
| NaH₂PO₄ · H₂O | – | 2.76 g | 2.76 g | – |
| EDTA | 5 μL of stock | – | – | – |
| Imidazole | – | – | 34.04 g | – |
| Titriplex III | – | – | – | 7.44 g |
| pH adjustment | HCl/NaOH to pH 7.3 | NaOH to pH 7.5 | NaOH to pH 7.5 | – |
| Final volume | ad 0.5 L | ad 1 L | ad 1 L | ad 0.1 L |

**Table S3.** Buffer compositions used for bud-scar staining and CFDA-based viability measurements.

| **Buffer** | **Composition** |
| --- | --- |
| MOPS buffer | 20 mM MOPS, pH adjusted to 4–7 with NaOH |
| ICP buffer | McIlvaine (citrate–phosphate) buffer: 50 mM Na₂HPO₄ and 50 mM citric acid, supplemented with 110 mM NaCl, 5 mM KCl, and 1 mM MgCl₂; pH adjusted to 3–6 |

**Table S4.** Parameters of Gaussian mixture models fitted to mCherry–ChBD (ECD-A) fluorescence distributions for all four yeast strains under ICP buffer conditions. Only fits with R² > 0 are reported. Component 1 corresponds to daughter cells; higher components represent successive mother-cell generations with increasing replicative age. μ: component mean ECD-A intensity; σ: standard deviation; w: component weight (proportion of total population); K: total number of fitted components; R²: goodness-of-fit.

| **Strain** | **pH** | **K** | **R²** | **Comp.** | **μ (ECD-A)** | **σ (ECD-A)** | **w** |
| --- | --- | --- | --- | --- | --- | --- | --- |
| *S. pastorianus* W-34/70 | 3 | 4 | 0.984 | 1 | 401.0 | 226.8 | 0.508 |
| *S. pastorianus* W-34/70 | 3 | 4 | 0.984 | 2 | 1142.5 | 523.0 | 0.247 |
| *S. pastorianus* W-34/70 | 3 | 4 | 0.984 | 3 | 3394.6 | 1579.3 | 0.134 |
| *S. pastorianus* W-34/70 | 3 | 4 | 0.984 | 4 | 10934.7 | 5557.7 | 0.111 |
| *S. pastorianus* W-34/70 | 4 | 5 | 0.996 | 1 | 326.3 | 188.2 | 0.612 |
| *S. pastorianus* W-34/70 | 4 | 5 | 0.996 | 2 | 904.4 | 393.1 | 0.187 |
| *S. pastorianus* W-34/70 | 4 | 5 | 0.996 | 3 | 1909.1 | 691.2 | 0.096 |
| *S. pastorianus* W-34/70 | 4 | 5 | 0.996 | 4 | 3935.2 | 1361.6 | 0.065 |
| *S. pastorianus* W-34/70 | 4 | 5 | 0.996 | 5 | 7874.5 | 2683.2 | 0.039 |
| *S. pastorianus* W-34/70 | 5 | 5 | 0.996 | 1 | 330.2 | 191.8 | 0.578 |
| *S. pastorianus* W-34/70 | 5 | 5 | 0.996 | 2 | 898.1 | 382.9 | 0.252 |
| *S. pastorianus* W-34/70 | 5 | 5 | 0.996 | 3 | 1831.7 | 668.7 | 0.076 |
| *S. pastorianus* W-34/70 | 5 | 5 | 0.996 | 4 | 3862.5 | 1379.6 | 0.063 |
| *S. pastorianus* W-34/70 | 5 | 5 | 0.996 | 5 | 7655.3 | 2768.7 | 0.032 |
| *S. pastorianus* W-34/70 | 6 | 5 | 0.997 | 1 | 312.6 | 189.9 | 0.647 |
| *S. pastorianus* W-34/70 | 6 | 5 | 0.997 | 2 | 812.6 | 273.4 | 0.238 |
| *S. pastorianus* W-34/70 | 6 | 5 | 0.997 | 3 | 1482.3 | 475.7 | 0.070 |
| *S. pastorianus* W-34/70 | 6 | 5 | 0.997 | 4 | 2626.3 | 856.1 | 0.035 |
| *S. pastorianus* W-34/70 | 6 | 5 | 0.997 | 5 | 4045.7 | 1499.0 | 0.010 |
| *K. phaffii* X33 | 3 | 3 | 0.951 | 1 | 1779.3 | 887.6 | 0.466 |
| *K. phaffii* X33 | 3 | 3 | 0.951 | 2 | 4742.0 | 2066.1 | 0.405 |
| *K. phaffii* X33 | 3 | 3 | 0.951 | 3 | 13000.9 | 6765.7 | 0.129 |
| *K. phaffii* X33 | 4 | 3 | 0.952 | 1 | 756.6 | 447.3 | 0.503 |
| *K. phaffii* X33 | 4 | 3 | 0.952 | 2 | 2445.7 | 1160.5 | 0.367 |
| *K. phaffii* X33 | 4 | 3 | 0.952 | 3 | 6331.1 | 3204.8 | 0.130 |
| *K. phaffii* X33 | 5 | 5 | 0.974 | 1 | 163.0 | 187.0 | 0.785 |
| *K. phaffii* X33 | 5 | 5 | 0.974 | 2 | 663.0 | 228.7 | 0.133 |
| *K. phaffii* X33 | 5 | 5 | 0.974 | 3 | 1260.0 | 402.6 | 0.050 |
| *S. cerevisiae var. diastaticus* BE-134 | 3 | 3 | 0.939 | 1 | 605.2 | 406.5 | 0.601 |
| *S. cerevisiae var. diastaticus* BE-134 | 3 | 3 | 0.939 | 2 | 2207.7 | 1182.3 | 0.290 |
| *S. cerevisiae var. diastaticus* BE-134 | 3 | 3 | 0.939 | 3 | 7521.7 | 4686.5 | 0.108 |
| *S. cerevisiae var. diastaticus* BE-134 | 4 | 3 | 0.956 | 1 | 575.3 | 385.3 | 0.557 |
| *S. cerevisiae var. diastaticus* BE-134 | 4 | 3 | 0.956 | 2 | 1818.0 | 934.8 | 0.319 |
| *S. cerevisiae var. diastaticus* BE-134 | 4 | 3 | 0.956 | 3 | 9545.3 | 6945.4 | 0.124 |
| *S. cerevisiae var. diastaticus* BE-134 | 5 | 3 | 0.933 | 1 | 836.2 | 566.2 | 0.586 |
| *S. cerevisiae var. diastaticus* BE-134 | 5 | 3 | 0.933 | 2 | 2751.1 | 1413.7 | 0.279 |
| *S. cerevisiae var. diastaticus* BE-134 | 5 | 3 | 0.933 | 3 | 10491.2 | 6516.9 | 0.136 |
| *S. cerevisiae var. chevalieri* LA-01 | 3 | 3 | 0.885 | 1 | 1218.7 | 744.0 | 0.515 |
| *S. cerevisiae var. chevalieri* LA-01 | 3 | 3 | 0.885 | 2 | 4414.3 | 2333.6 | 0.248 |
| *S. cerevisiae var. chevalieri* LA-01 | 3 | 3 | 0.885 | 3 | 26531.5 | 15978.2 | 0.237 |
| *S. cerevisiae var. chevalieri* LA-01 | 4 | 3 | 0.953 | 1 | 1026.7 | 566.5 | 0.497 |
| *S. cerevisiae var. chevalieri* LA-01 | 4 | 3 | 0.953 | 2 | 2883.1 | 1335.6 | 0.412 |
| *S. cerevisiae var. chevalieri* LA-01 | 4 | 3 | 0.953 | 3 | 9755.7 | 5841.1 | 0.092 |
| *S. cerevisiae var. chevalieri* LA-01 | 5 | 3 | 0.962 | 1 | 802.8 | 452.2 | 0.494 |
| *S. cerevisiae var. chevalieri* LA-01 | 5 | 3 | 0.962 | 2 | 2563.6 | 1204.0 | 0.418 |
| *S. cerevisiae var. chevalieri* LA-01 | 5 | 3 | 0.962 | 3 | 7042.8 | 3918.3 | 0.088 |

**Supplementary Figures**

**Figure S1.** Threshold calibration for CFDA-based viability classification. The log₁₀(CFDA/PerCP) distribution at pH 3 is shown for all four strains. Dashed lines indicate the fixed thresholds applied per strain group (CFDA/PerCP ratio = 0.7 for *S. pastorianus* W-34/70 and *K. phaffii* X33; ratio = 10.0 for *S. cerevisiae* var. diastaticus BE-134 and *S. cerevisiae* var. chevalieri LA-01), each positioned at the bimodal valley of the respective distribution.

**Figure S2.** Extended gating strategy for MOPS buffer at pH 4 and pH 5. Representative FSC-H versus PC7-H gating plots illustrating the progressive two-step filtering workflow applied under conditions of elevated PC7-H autofluorescence. Raw events (A) were subjected to an extended rectangular pre-gate (B), followed by Mahalanobis distance-based outlier removal (C). Ellipses indicate the 95% and 99.9% confidence regions used for Mahalanobis gating. The override gate retains the full event population that would otherwise be lost under the default PC7-H threshold.

**Figure S3.** CFDA-based viability discrimination in *K. phaffii* X33 as a function of buffer composition and pH. Mean viable fraction ± SD across three biological replicates is shown for ICP and MOPS buffer across the tested pH range. ICP buffer at pH 3–5 provides stable and reproducible viability discrimination. MOPS buffer yields unreliable and highly variable estimates across all pH values tested. CFDA measurements were restricted to cultures in active exponential growth phase (OD₆₀₀ = 4.5 ± 0.3).

**Figure S4.** Validation of CFDA-based viability measurement under simultaneous dual-parameter staining conditions in *S. pastorianus* W-34/70. (A) Population-level viability as a function of pH for single CFDA staining (CFDA only, red) and simultaneous mCherry–ChBD and CFDA double staining (CFDA + mCherry, blue) in ICP buffer. Points indicate individual biological replicates; lines show mean values with shaded areas representing ± SD. (B) Paired comparison of viability estimates from single versus double staining per pH and replicate. The dashed line indicates perfect agreement (y = x); the red line shows the linear regression fit (slope = 0.78, R² = 0.20). The close agreement between single and double staining conditions confirms that mCherry–ChBD labeling does not substantially interfere with CFDA-based viability discrimination across pH 3–5.

**Figure S5.** Age-class-specific metabolic viability in all four yeast strains under ICP buffer conditions, displayed as line plots. Viable fraction per Gaussian mixture model component is shown for each tested pH value. Component 1 corresponds to daughter cells; higher components represent successive mother-cell generations. Error bars indicate standard deviation across three biological replicates.

**Figure S6.** Confocal laser scanning microscopy of *S. cerevisiae* var. diastaticus BE-134 cells under simultaneous dual-parameter staining in ICP buffer at pH 4. Green channel: CFDA-derived fluorescence in metabolically active cells with intact membranes. Red channel: mCherry fluorescence localized at bud-scar structures on the mother-cell surface. Grey channel: brightfield transmission. Right: Overlay showing the total cell population. Cells showing both green and red fluorescence represent viable mother cells carrying at least one bud scar. Companion montage to Figure 7 (*S. pastorianus* W-34/70) and Supplementary Figure S7 (*S. cerevisiae* var. chevalieri LA-01). Scale bar: 20 μm.

**Figure S7.** Confocal laser scanning microscopy of *S. cerevisiae* var. chevalieri LA-01 cells under simultaneous dual-parameter staining in ICP buffer at pH 4. Green channel: CFDA-derived fluorescence in metabolically active cells with intact membranes. Red channel: mCherry fluorescence localized at bud-scar structures on the mother-cell surface. Grey channel: brightfield transmission. Right: Overlay showing the total cell population. Cells showing both green and red fluorescence represent viable mother cells carrying at least one bud scar. Companion montage to Figure 7 (*S. pastorianus* W-34/70) and Supplementary Figure S6 (*S. cerevisiae* var. diastaticus BE-134). Scale bar: 20 μm.
